# Proteomic and Transcriptomic Analyses in the Slipper Snail *Crepidulafornicata* Uncover Shell Matrix Genes Expressed During Adult and Larval Biomineralization

**DOI:** 10.1093/iob/obac023

**Published:** 2022-08-10

**Authors:** G O Batzel, B K Moreno, L S Lopez, C K Nguyen, B T Livingston, D Joester, D C Lyons

**Affiliations:** Center for Marine Biotechnology and Biomedicine, Scripps Institution of Oceanography, UCSD, La Jolla, CA 92037, USA; Department of Materials Science and Engineering, Northwestern University, Evanston, IL 60208, USA; Department of Biological Sciences, California State University, Long Beach, CA 90802, USA; Department of Biological Sciences, California State University, Long Beach, CA 90802, USA; Department of Biological Sciences, California State University, Long Beach, CA 90802, USA; Department of Materials Science and Engineering, Northwestern University, Evanston, IL 60208, USA; Center for Marine Biotechnology and Biomedicine, Scripps Institution of Oceanography, UCSD, La Jolla, CA 92037, USA

## Abstract

The gastropod shell is a composite composed of minerals and shell matrix proteins (SMPs). SMPs have been identified by proteomics in many molluscs, but few have been studied in detail. Open questions include (1) what gene regulatory networks regulate SMP expression, (2) what roles individual SMPs play in biomineralization, and (3) how the complement of SMPs changes over development. These questions are best addressed in a species in which gene perturbation studies are available; one such species is the slipper snail, *Crepidula fornicata*. Here, SEM and pXRD analysis demonstrated that the adult shell of *C. fornicata* exhibits crossed lamellar microstructure and is composed of aragonite. Using high-throughput proteomics we identified 185 SMPs occluded within the adult shell. Over half of the proteins in the shell proteome have known biomineralization domains, while at least 10% have no homologs in public databases. Differential gene expression analysis identified 20 SMP genes that are up-regulated in the shell-producing mantle tissue. Over half of these 20 SMPs are expressed during development with two, CfSMP1 and CfSMP2, expressed exclusively in the shell gland. Together, the description of the shell microstructure and a list of SMPs now sets the stage for studying the consequences of SMP gene knockdowns in molluscs.

## Introduction

The ability to create biominerals is found in all kingdoms of life, ranging from the siliceous frustules of single-celled diatoms to the internal calcium phosphate skeletons of birds and mammals (Blakemore 1975; Noll et al. 2002; Hu et al. 2010; Leão et al. 2020; Liu et al. 2021). The earliest known mineralized structures produced by organisms date back 3.3–3.5 billion years ago to stromatolites discovered in shallow basins of Western Australia (Schopf and Packer 1987; Riding 1991). The biominerals produced by stromatolites, and by many lithifying bacteria present today, are examples of biologically induced mineralization, in which advantageous precipitation of minerals occurs either as a byproduct of metabolism, or charged cell walls that come in contact with the environment (Douglas and Beveridge 1998; Dupraz and Visscher 2005). This contrasts with the evolution of biologically-controlled deposition of minerals particularly in animals, which is a genetically controlled process by which specialized cells and tissues secrete extracellular matrix (ECM) proteins that direct the growth of mineral structures. Mineralized tissues have evolved diverse biological functions such as use for housing, locomotion, feeding, protection, and sensing (Mann 1983; Lowenstam and Weiner 1989; Stegbauer et al. 2021; Varney et al. 2021). The current phylogenetic distribution of carbonate skeletons in animals suggests that carbonate biomineralization has evolved independently at least 20 times in animals (Knoll 2003); yet, a standing question is to what degree the molecular pathways and gene regulatory networks (GRNs) underlying biomineralization are conserved across all animals that produce biominerals (Ettensohn 2009). These evolutionary comparisons will first require greater mechanistic understanding of biomineralization processes in a diverse sampling of animals. Genetic tools can be used to dissect the function of genes controlling skeletogenesis, and have resulted in greater mechanistic insight into vertebrate biomineralization, particularly in mice and zebrafish (Wilt 2005). However, functional studies in non-model species, primarily among invertebrates, are few and far between, relative to vertebrates.

The Mollusca are the second most speciose phylum of metazoans, and have undergone extensive diversification in their biomineral structures. The molluscan shell is constructed from an extracellular organic matrix composed of shell matrix proteins (SMPs), polysaccharides (Marxen et al. 1998), and lipids (Farre and Dauphin 2009). These macromolecules contribute to the mechanical properties of the shell and help direct the growth of mineral structures at the molecular level (Marin and Luquet 2004). The most well studied of the organic matrix macromolecules are SMPs (Aguilera et al. 2017), and to date, SMPs have been identified through proteomic and/or transcriptomic approaches in over 55 species of molluscs (Marin 2020). By comparing SMPs between different molluscan species, it has been observed that SMPs have undergone repeated independent expansions in different molluscan lineages, leading to speculation that lineage-restricted SMPs may underlie shell diversity (color, shape, pattern, and ultrastructure) (Kocot et al. 2016). These lineage-restricted SMPs often show signs of intrinsic disorder or low complexity regions, which are regions of a protein that undergo fast evolutionary rates and do not undergo conformational folding unless bound to a substrate or under the right physiological conditions (Dyson and Wright 2005). On the other hand, these same studies revealed that molluscan shell proteomes share some similarities, for example, having clear homologs of certain protein families involved in regulating calcium, or having proteins that harbor highly conserved protein domains (e.g., ECM-binding domains) that might have been acquired through domain shuffling between otherwise non-homologous proteins (Kocot et al. 2016). SMPs–or the protein domains therein–that are shared between species might serve fundamental roles in shell construction and integrity (Marin 2020).

Such hypotheses about the function of SMPs remain largely untested because relatively few molluscan SMPs have been studied in detail, and even fewer have been functionally tested via gene perturbation experiments (Jackson and Degnan 2016). Thus, while “omics” approaches have generated comprehensive lists of molluscan SMPs, fundamental questions remain. For example: When and where are SMPs first expressed during larval shell gland development, and does the complement of larval SMPs undergo extensive GRN rewiring during adult shell formation? How are SMPs transcriptionally regulated so that they are expressed in cell lineages that give rise to the larval shell gland, and later to the adult mantle? What is the functional consequence for the larval or adult biomineral, if any, of removing or down-regulating specific SMPs? To answer these and related questions, an experimental species is necessary in which embryonic material is accessible, and in which it is possible to deliver reagents for gene perturbation to their embryos. The marine slipper snail *Crepidula fornicata* is one such species (Perry and Henry 2015), and thus is an ideal candidate for studying the regulation and function of SMPs in the larval and adult shell.

A member of the Caenogastropoda, a subclass of gastropods that all share aragonitic and crossed-lamellar microstructure shells (Ponder et al. 2008), *C.fornicata* is one of the most experimentally tractable molluscan systems for studying developmental biology (Henry, Collin, et al. 2010; Henry and Lyons 2016). A number of tools and genomic resources are available, including a detailed embryonic staging system (Henry, Collin, et al. 2010; Henry and Lyons 2016; Lyons and Henry 2022), high resolution cell-lineage fate maps (Hejnol et al. 2007; Lyons et al. 2012, 2015, 2017), embryonic transcriptomes (Henry, Perry, Fukui, et al. 2010), and gene perturbation tools (Henry, Perry, and Martindale 2010; Perry and Henry 2015). Furthermore, key details are already known about shell development. For example, the larval shell gland is derived from all eight second-quartet daughter cells (2a1/2a2, 2b1/2b2, 2c1/2c2, 2d1/2d2) (Hejnol et al. 2007; Lyons et al. 2015). Additionally, *in-situ* hybridization studies identified transcription factors expressed in the larval shell gland (Perry et al. 2015; Osborne et al. 2018; Lyons et al. 2020). These data provide a preliminary set of “upstream” transcription factors that may regulate the transcription of “downstream” terminal differentiation effector genes, such as SMPs. The next challenge for studying shell formation in *C. fornicata* is to perturb gene function to confirm not only the cis-regulatory relationships between transcription factors and SMPs, but also to test the function of specific SMPs themselves. For example, gene perturbation experiments targeting transcription factors can be carried out and the expression of downstream effector genes can be used as phenotypes (either through *in-situ* hybridization or other means of assessing mRNA abundance like qPCR). In order to perform these types of experiments, an extensive understanding of the SMP complement of the shell is required to identify downstream genes within mantle-specific GRNs. With these candidate SMPs in hand, gene perturbation experiments will require an analysis of the shell microstructure to compare against wildtype shells, as well as a list of candidate SMPs to begin to perturb. This study identifies the adult shell microstructure, its SMP composition, and identifies two specific SMPs that are exclusively expressed in the shell gland in *C. fornicata*.

## Results

### Adult shells are composed of aragonite and exhibit crossed-lamellar microstructure

**Fig. 1:**
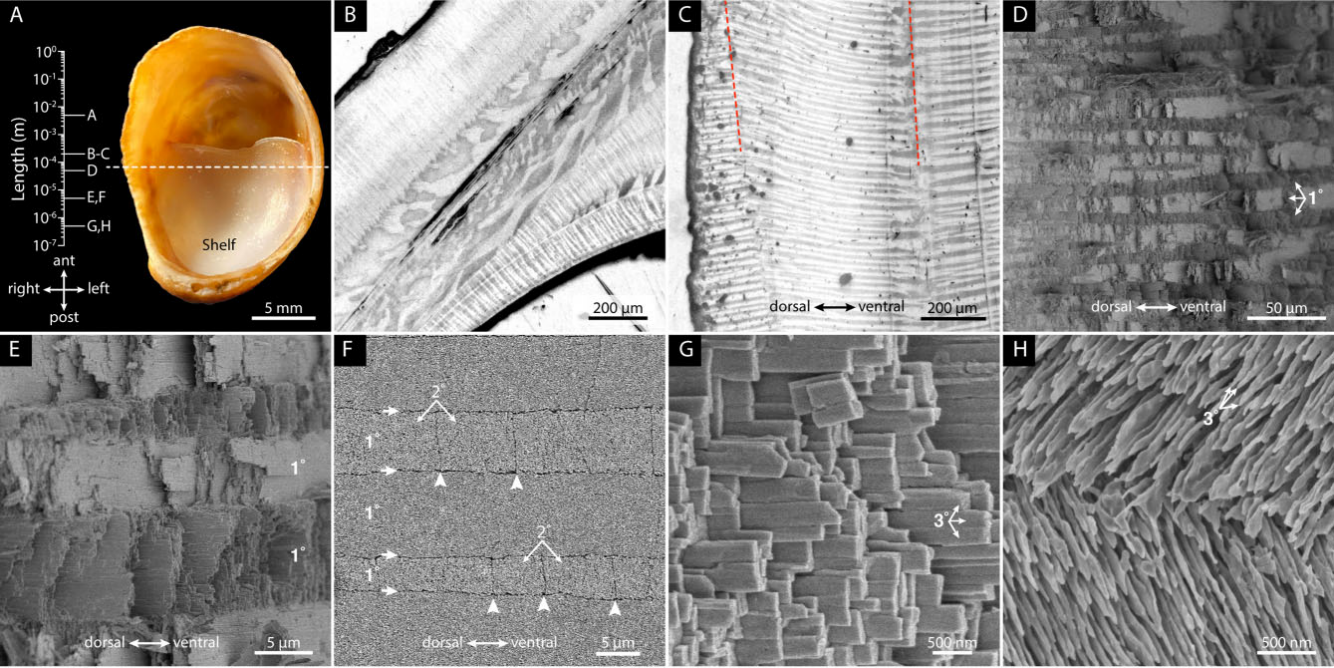
Length scales of the crossed-lamellar microstructure of the shell of *C. fornicata*. A: Ventral view of the adult shell. B–C: Reflected, polarized light microscopy images of sections approximately normal to the anterior-posterior axis. A number of macro layers with different orientations of 1° lamellae are visible where the shelf is joined to the shell (B); on the arc between these junctions three macro layers can be recognized by the slightly different orientation of the banding pattern (dashed lines, C). D: SEM image of a fracture surface, approximately normal to the anterior-posterior axis. White arrows indicate first order (1°) lamellae. E: Higher magnification image from the same area in B. F: SEM image of a polished and etched surface on the plane normal to the posterior-anterior axis and looking down the posterior direction. White arrows indicate boundaries between 1° lamellae and white arrowheads indicate boundaries between second order (2°) lamellae. G: SEM image of third order (3°) lamellae (white arrows) in a fracture surface. H: SEM image showing 3° lamellae (white arrows) in neighboring 1° lamellae in a polished and etched sample.

To characterize the nature of the biomineral and the microstructural organization of the adult shell (Fig. S1), we used powder X-ray diffraction (PXRD) and scanning electron microscopy on adult shells of *C. fornicata* (Fig. 1A). PXRD patterns matched those of a geological reference material and literature data (Bouasria et al. 2021), confirming that the calcium carbonate polymorph in *C. fornicata* shells is aragonite (Fig. S2). Fracture surfaces and sections of *C. fornicata* shells reveal a hierarchical organization typical of the crossed-lamellar shell structure. When viewed between crossed polarizers, the orientation of the crystal lattice with respect to the polarizers determines how bright or dark single crystals of birefringent aragonite appear. For polycrystalline sections of level thickness, the brightness depends on the local orientation of the lattice; lattice orientation thus gives rise to contrast in the polarized light image. In adult *C. fornicata* shells, macro layers parallel to the shell surface displayed alternating bands of light and dark contrast, consistent with linear and branched first-order (1°) lamellae (Fig. 1B) (Carter and Clark 1985). Interestingly, 1° lamellae at the outer surface appeared to be oriented normal to the shell surface in several consecutive macro layers (Fig. 1B–C). In SEM images of fracture surfaces, first-order (1°) lamellae appear as alternating dark and light-colored bands 5–20 µm in width (Fig. 1D–E). The 1° lamellae are comprised of second order (2°) lamellae that give rise to the characteristic stepped fracture surface. In ground, polished, and lightly etched sections, 2° lamellae are distinguished by their linear boundaries that run perpendicular to the boundaries of the 1° lamellae (Fig. 1F). At higher magnification, the third order (3°) lamellae are visible as stacked aragonite laths 100–250 nm in thickness (Fig. 1G). Etching partially dissolves the 3° lamellae into thin aragonite needles and makes their orientation more apparent, such as the ∼90° misorientation between 3° lamellae in neighboring 1° lamellae (Fig. 1H).

### The adult shell proteome of *C. fornicata* comprises at least 185 SMPs

To identify SMPs in adult shells of *C. fornicata*, we performed high throughput proteomics and next generation RNA sequencing on shells and mantle, respectively. First, the soluble and insoluble proteins occluded in the shell organic matrix were isolated and separated by SDS-PAGE. Gel lanes were divided into 20 sections and each was analyzed separately. After tryptic digestion, peptides were analyzed by liquid chromatography tandem mass spectrometry (LC-MS/MS) and compared against a six-frame translated mantle transcriptome (Fig. S3). In total, 7056 spectra were recovered, yielding 1617 unique peptides. We applied a 95% protein probability with a false discovery rate of < 1% to our tandem mass spectra. Using a minimum of two peptide hits for each protein of 50 amino acids or greater, we were able to identify 185 SMPs (Table S1, S2): 55 (29%) SMPs were found in both soluble and insoluble fractions; 84 (45%) were found only in soluble fractions; and 45 (24%) were found only in insoluble fractions.

### Half of the shell proteome contains functional domains implicated in biomineralization

To characterize SMPs from *C. fornicata* and to compare them to other species, we conducted annotations on the protein coding sequences for all 185 SMPs (Fig. S3; Table S3). First, all 185 SMPs were examined for coding regions using the program TransDecoder, which identified complete open reading frames (ORFs) in 97 of 185 (52%) SMPs (Table S1–S3). Next, all 185 SMPs underwent BLASTP searches (e-value < 10e-6) against the non-redundant protein sequence database (NCBI) to find regions of similarity between *C. fornicata* SMPs and publicly available sequences. In total, 131 of 185 (71%) SMPs had BLASTP hits (Table S1–S3). To identify functional domains in our SMPs, hidden Markov model (HMMER) searches were performed using SMP coding sequences against the Pfam domain database (e-value < 10e-3; Bateman et al. 2004). In total, 102 of 185 (55%) SMPs had at least one identifiable functional domain in their sequence (Figure S3; Table S3). Annotations were made for all 185 SMPs using their best BLAST hit and Pfam domain descriptions (Supplementary Note 1). Each SMP was placed into one of six previously defined categories of molluscan SMPs (Marin 2020): (1) ECM binding proteins, (2) calcium binding and signaling proteins, (3) proteases and inhibitors, (4) intrinsically disordered (ID), (5) enzymatic, and (6) putative lineage-restricted and uncharacterized proteins (Table S1, S2; Supplementary Note 1).

### ID proteins make up 39% of the shell proteome

**Fig. 2:**
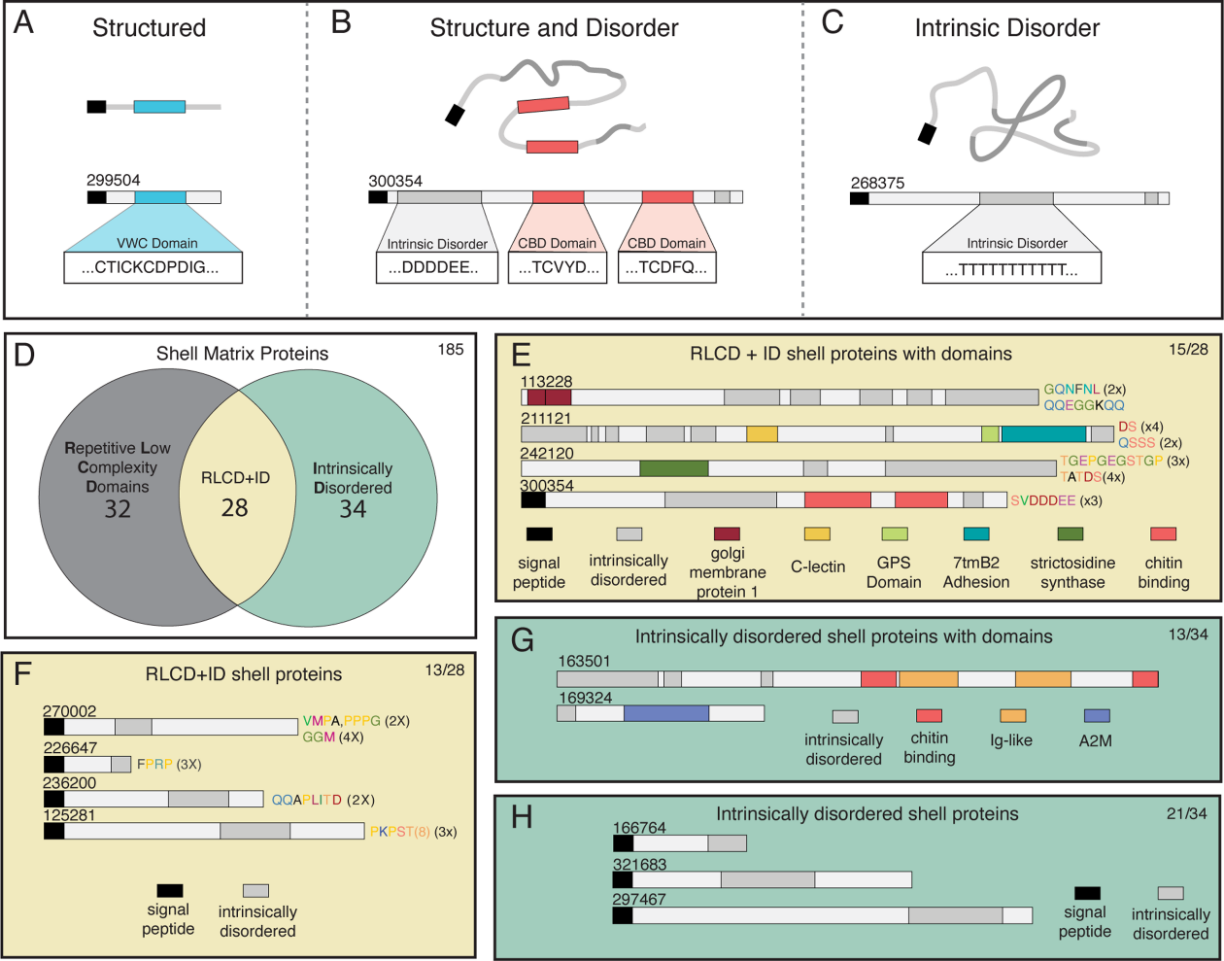
Intrinsically disordered shell matrix proteins in *C. fornicata*. A–C: Three categories of structured and disordered SMPs. These three categories are illustrated using SMPs found from the adult shell proteome of *C. fornicata*. A: An example from our shell proteome of a structured protein containing a functional domain, but no intrinsic disorder (ID) or repetitive low complexity (RLCD) domains. In this example, the blue box indicates a von Willebrand type C domain, while the black box indicates signal peptide domain. B: Structure and disordered proteins may also have functional domains as well as ID and RLCD regions. For example, gray boxes are regions of ID/RLCD that do not undergo conformational folding, while red boxes are examples of chitin binding domains found within protein sequences containing ID/RLCD domains. C: An example of intrinsically disordered proteins with no conserved domains. Note the absence of structured functional domains in this example sequence. D: Venn diagram comparison of SMPs from *C. fornicata* that contain regions of RLCD, ID, or both RLCD and ID domains. E: Examples of SMPs from *C. fornicata* that have RLCD, ID, and functional domains. F: Examples of SMPs from *C. fornicata* with RLCD and ID domains, but no functional domains. G: Examples of SMPs from *C. fornicata* with ID and Pfam domains, but no RLCDs. H: Examples of SMPs from *C. fornicata* with ID domains, but no RLCD or functional domains.

The largest category of SMPs in the shell proteome consisted of SMPs with regions of intrinsic disorder (Fig. 2). ID proteins possess regions of their coding sequence that either lack secondary structure, or do not fold into a stable tertiary structure (McDougall et al. 2013; Kocot et al. 2016). Some ID proteins contain known domains interspersed between ID regions, leading to partially folded proteins (Van Der Lee et al. 2014) (Fig. 2A–B). Entire protein sequences may lack any folded structure due to ID regions spanning entire ORFs (Van Der Lee et al. 2014) (Fig. 2C). Two programs were used to identify regions of intrinsic disorder in *C. fornicata* SMPs: IUPred (Dosztányi et al. 2005) examined inter-residue interaction energy in protein folding and stability, while XSTREAM (Newman and Cooper 2007) identified short tandem repeats, which are highly correlated and often found within ID regions in proteins (Delucchi et al. 2020). IUPred identified 34 SMPs with ID regions (Fig. 2D; (Table S1–S3), while XSTREAM found 60 SMPs with Repetitive Low Complexity Domains (RLCD) (Fig. 2D; Table S1–S3). RLCD and ID domains were both identified in 28 SMPs (Fig. 2D). All SMPs with either RLCD or ID domains were searched against the Pfam domain database for the presence of conserved protein domains: 15 SMPs had at least one other conserved domain (Fig. 2E), while 13 SMPs had no conserved domains (Fig. 2F). Based on sequences with only ID features, 13 SMPs had ID regions with at least one conserved domain (Fig. 2G), while the remaining 21 SMPs only had ID regions (Fig. 2H).

### Differential expression reveals 39 genes upregulated in the mantle

**Fig. 3:**
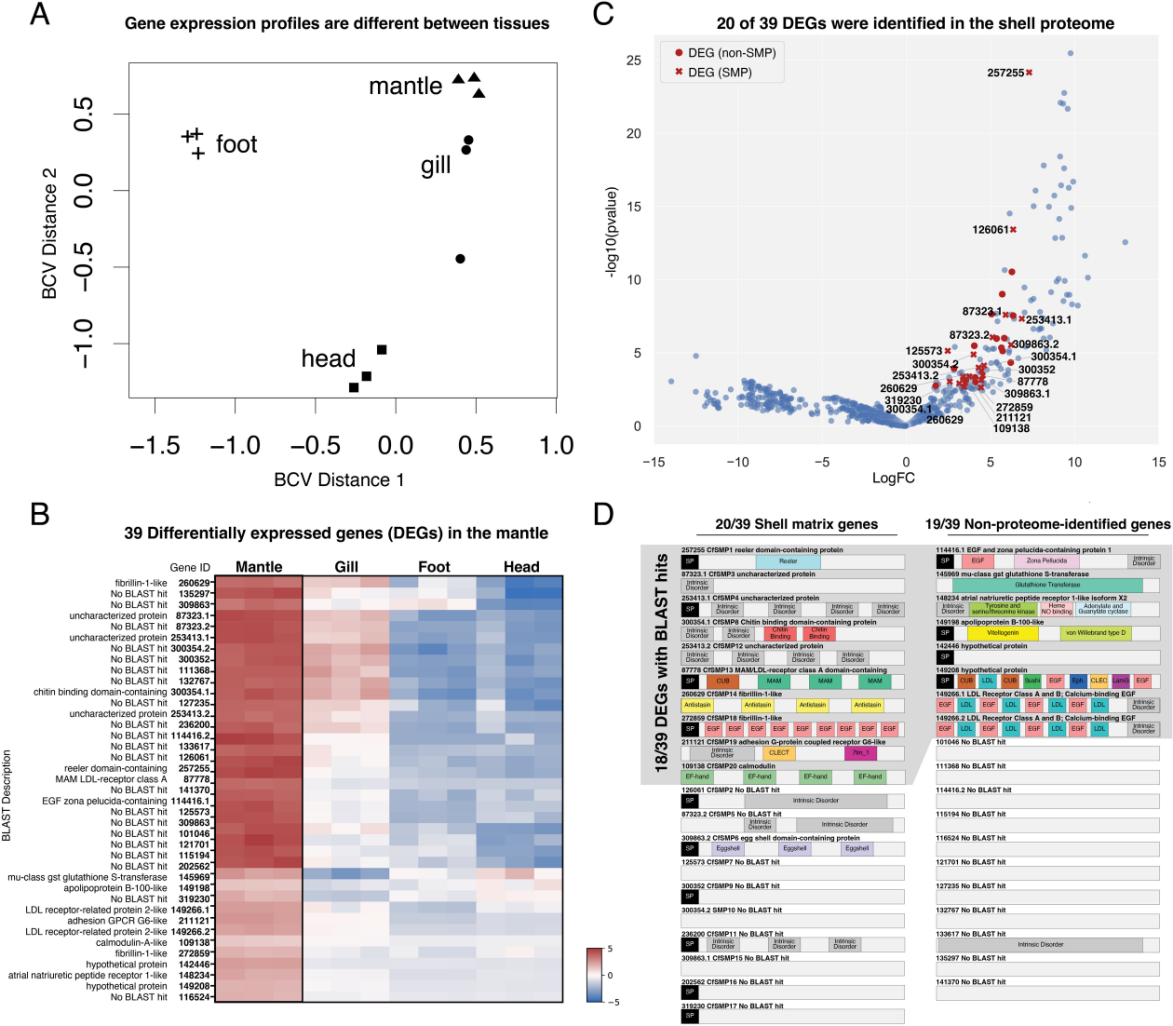
Differentially expressed genes in the adult mantle of *C. fornicata*. A: Multidimensional scaling (MDS) plot showing gene expression similarities between tissue types. Greater distance between points indicate samples that are less similar. B: Heatmap of 39 differentially expressed genes in the adult mantle. Genes are represented in rows, while columns represent tissue samples and their biological replicates. Overexpressed genes are red, while underexpressed genes are blue. C: Volcano plot showing significantly differentially expressed genes in the mantle compared to head, gill, and foot combined. Red denotes 39 differentially expressed mantle genes. D: Gene schematics for all 39 differentially expressed genes in the mantle.

The shell proteome provided a candidate list of adult SMPs to screen during larval shell development. To prioritize which adult SMP to screen during embryogenesis, differential expression analysis was performed for the adult mantle (organ that is responsible for secreting SMPs; referred to as a tissue or collection of tissues at various times in the text), and compared against non-biomineralizing organs such as the head, foot, and gill. Four pairwise comparisons (head, foot, gill, and mantle) were conducted, with three biological replicates for each condition. Gene expression profiles were significantly different between conditions, while biological replicates for each condition were similar (Fig. 3A). In total, 39 transcripts were found significantly (FDR corrected *P*-value ≤ 0.001; Log2FC ≥ 4), differentially expressed in the mantle compared to three other tissue types combined (Fig. 3B; Figure S4; Table S4). Of the 39 differentially expressed genes in the mantle, 20 were SMPs identified from the adult shell proteome, while 19 transcripts were non-proteome-identified genes (Fig. 3C–D; Figure S5, S6; Supplementary Note 2). The 20 differentially expressed SMPs were named *C.fornicata* Shell Matrix Protein 1 through 20 (CfSMP1-20), based on their logFC values ranging from highest (CfSMP1) to lowest (CfSMP20).

### Ten differentially expressed adult SMPs are expressed in the larval shell gland

**Fig. 4:**
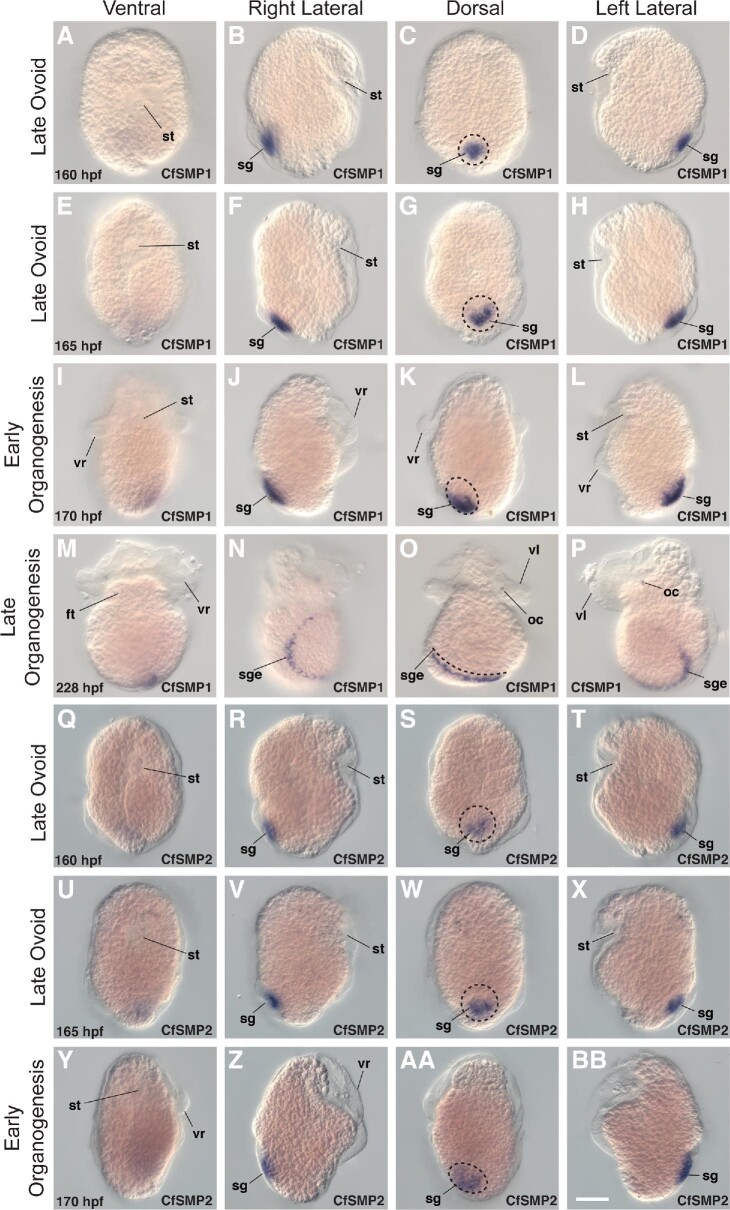
CfSMP1 and CfSMP2 expression during larval development in *C. fornicata*. A–P: CfSMP1 expression in embryos between 160–228 hpf. Q-BB: CfSMP2 expression in embryos between 160–180 hpf. All animals oriented with anterior up and posterior down. Column panels show different embryo orientations, while panel rows show different developmental stages. A–D: CfSMP1 expression in the shell gland in late ovoid embryos (160 hpf). E–H: Expression persists in the shell gland (165 hpf). I–L: In early organogenesis staged embryos (170 hpf), shell gland expression of shifts left of the midline. M–P: By late organogenesis (228 hpf), CfSMP1 is expressed in the shell gland edge. Q–T: CfSMP2 expression in the shell gland in late ovoid embryos (160 hpf). U–X: Expression persists in the shell gland in late ovoid embryos (165 hpf). Y-BB: Shell gland expression is apparent in early organogenesis staged embryos (170 hpf). Structure labels: st, stomodeum; hr, hindgut rudiment; sg, shell gland; sge, shell gland edge; vr, velar rudiment; vl, velar lobe; oc, ocelli; ft, foot. Scale bar = 50 μm.

Previous larval shell proteomic studies found few SMPs that are present in both the larval and adult shell, suggesting different repertoires of larval and adult SMPs (Zhao et al. 2018; Carini et al. 2019). We hypothesized that of the 185 SMPs identified in the adult shell proteome of *C. fornicata*, few were likely to be expressed during larval shell development. Instead, we asked whether any of the most differentially expressed SMPs in the adult mantle were also expressed in the larval shell gland. Primers were designed for all 20 SMP sequences (Table S5), and ten SMPs were successfully amplified. These ten differentially expressed SMPs (CfSMP1, CfSMP2, CfSMP3, CfSMP5, CfSMP9, CfSMP10, CfSMP12, CfSMP14, CfSMP17, CfSMP20; Table S4) were examined by whole mount *in-situ* hybridization (WMISH) during larval shell development, including five genes with BLAST hits in GenBank, and five without (Fig. 3D; Figure S7; Table S4). All ten genes were determined to be expressed in the larval shell gland. Two SMPs (CfSMP1 and CfSMP2) were exclusively expressed in shell gland cells (Fig. 4), while the remaining eight SMPs (CfSMP3, CfSMP5, CfSMP9, CfSMP10, CfSMP12, CfSMP14, CfSMP17, CfSMP20) were expressed in multiple embryonic tissues including the shell gland (Figure S7–S17; Supplementary Note 3). Detailed results for all 10 genes can be found in the Supplementary, including a detailed description of shell gland induction in *C. fornicata* (Fig. S8–S17; Supplementary Note 1–3).

### CfSMP1 and CFSMP2 are restricted to the larval shell gland during development

We successfully identified two SMPs that are expressed exclusively in the shell gland during development. CfSMP1 was the most differentially expressed SMP (4.61 logFC) (Fig. 4A–P; Fig. 3B–C; Table S4). The 825 base pair (275 amino acid) nucleotide sequence contains a complete ORF, and has query coverage of 95% to a hypothetical protein from the slug, *Elysia chlorotica* (RUS86933), but low overall percent identity (26.67%). The majority of BLAST hits to CfSMP1 align to a 113 amino acid region in CfSMP1 that encodes a Reeler domain, which is an ECM binding domain (Hirotsune et al. 1995). CfSMP1 also contains a 45 amino acid region of intrinsic disorder that ends before the stop codon, and consists primarily of glycine (33%) and glutamine (22%). The second shell gland-restricted gene that we identified was CfSMP2 (Fig. 4Q–BB), the second most differentially expressed SMP (4.46 logFC) in the adult mantle (Fig. 3B–D; Table S4). CfSMP2 is a 945 bp (315 aa) nucleotide sequence and encodes a complete ORF (Fig. 3D). CfSMP2 returned no BLAST hit in GenBank, and returned no Pfam functional domains. Instead, CfSMP2 contains a 128 amino acid region of intrinsic disorder that makes up 41% of its protein coding sequence. Furthermore, CfSMP2 is composed primarily of the hydrophobic amino acids proline (21%) and glycine (9%).

CfSMP1 and CfSMP2 were the only shell gland-restricted SMPs that we identified out of 10 differentially expressed SMPs that were screened (Fig. 4; Fig. S7). Expression of CfSMP1 and CfSMP2 in the shell gland was present in mid ovoid staged embryos (160–170 hpf) (when the shell gland is first forming), and persisted in the shell gland through organogenesis (Fig. 4). During mid and late ovoid stages (160–170 hpf), CfSMP1 and CfSMP2 are restricted to the posterodorsal surface within the invaginated shell gland (Fig. 4A–H; Fig. 4Q–X). By 180 hpf, CfSMP1 and CfSMP2 expression in the shell gland shifts left of the midline on the posterodorsal surface (Fig. 4I–L; Fig. 4Y–BB). The invaginated shell gland later evaginates to form the shell field, and CfSMP1 expression is present in cells lining the marginal edge of the shell field (Fig. 4M–P).

## Discussion

### The microstructure and composition of the *C. fornicata* shell are typical for caenogastropods

Shells of *C. fornicata* were determined to be similar to that of other gastropod species at both a shell microstructure level and at a shell matrix protein level. For example, the most common shell microstructure in gastropods is the crossed lamellar structure (Boggild 1930; Wilmot et al. 1992). Similar to nacre, but less studied, it is composed of aragonite and a small fraction (<1% w/v) of organic matter (Dauphin et al. 2012; Li et al. 2017; Agbaje et al. 2019). The *C. fornicata* shell is composed of aragonite and PXRD revealed no indication for the presence of other crystalline minerals. The microstructure and composition of adult shells provide a reference to which shells from individuals subjected to perturbation experiments can be compared. This type of analysis would also be interesting to conduct on the veliger shell to determine when the transition from amorphous calcium carbonate to aragonite occurs during larval shell development. Future studies may look more closely at the larval shell microstructure development through time, which may prove to be an earlier and more interesting phenotype to target for gene perturbation studies, especially one that does not require growing the individual to adulthood.

From a shell proteomics perspective initial studies of molluscan shell proteomes identified a suite of SMPs (Aguilera et al. 2014; Le Roy et al. 2014; Jackson and Degnan 2016), and functional domains (Aguilera et al. 2017; Arivalagan et al. 2017) that are shared among molluscs. In line with this observation, at least 52% of the shell proteome of *C. fornicata* is comprised of SMPs with BLAST hits, including many previously identified functional domains that have been found in other molluscan SMPs, specifically: ECM binding domains (chitin binding, EGF, SPARC, Sushi), calcium-binding domains (EF hand and Ependymin), protease and inhibitor domains (Kunitz, CD109, IgG, Lipocalin), and enzymatic domains (glycoside hydrolase 18) (McDougall and Degnan 2018; Marin 2020). Previously identified matrix protein homologs were found such as Galaxin, which was originally identified in a coral skeletal proteome (Fukuda et al. 2003). We also identified calcium-binding proteins including calmodulin, calreticulin, and calumenin, which have roles in binding calcium ions in the ECM. These data indicate that at least 185 SMPs were identified in the adult shell of *C. fornicata*; however, this number likely represents a minimum number of proteins, as different methods for shell matrix protein extraction and sequencing can result in varying numbers of identified SMPs (Mann et al. 2012; Mann and Edsinger 2014; Arivalagan et al. 2017). Future studies might employ different shell cleaning conditions and proteomics methods to fully capture the complement of SMPs in the adult shell.

### At least 10% of *C. fornicata's* SMPs are “lineage-restricted”

Increased attention has been paid to lineage-restricted SMPs, which by the strictest definition share no sequence similarity or functional domains to previously characterized genes (Khalturin et al. 2009). We use the term lineage-restricted SMP to refer to proteins in the shell proteome of *C. fornicata* that have no BLAST hit or Pfam protein domains, and therefore may be genes that are only found, or restricted, at the *Crepidula* genus or species level. Some studies have speculated that lineage-restricted SMPs may be responsible for shell morphological characteristics, including their shape, pigmentation, or microstructure (Suzuki et al. 2009; McDougall and Degnan 2018). The shell proteome of *C. fornicata* contains at least 18 lineage-restricted SMPs, of which four were differentially expressed in the mantle. One of these genes, CfSMP2, was expressed only in the shell gland during development. The categorization of an SMP as lineage-restricted should be made after careful consideration of multiple factors. First, it is important to note that designation as a lineage-restricted SMP is relative to available sequences in public databases against which to compare. BLAST searches for CfSMP2 against the transcriptome from the closely related species, *Crepidula atrasolea*, whose sequences are not in GenBank, returned a BLAST hit that shares 59% identity to CfSMP2 (publication currently in preparation). This result demonstrates that as more sequencing data become available in public databases, the level at which an SMP is lineage-restricted may change; in this case, CfSMP1 would no longer be restricted at the species-level, but would still be considered lineage-restricted at the genus level. Second, BLAST searches of public databases often result in short alignments between two sequences that align around conserved protein domains, and require further phylogenetic analyses of the gene family to determine whether a gene is lineage-restricted. For example, BLAST searches for CfSMP1 against GenBank returned a hypothetical protein from *E.chlorotica* (RUS86933) that shared only 29% identity, concentrated primarily around the reeler domain—an ECM binding domain originally found in the neuronal gene Reelin and recently reported in the larval shell proteome of the bivalve *Mytilus edulis* (Carini et al. 2019). The short alignment between sequences in GenBank and CfSMP1 suggests that CfSMP1 could be a lineage-restricted SMP; however, like CfSMP2, we found a putative CfSMP1 homolog in *C. atrasolea* that shared 61.4% identity (data not shown). Given that the short alignments to CfSMP1 in GenBank centered around the reeler domain, and the identification of a putative CfSMP1 homolog in *C. atrasolea*, we hypothesize that CfSMP1 may have been co-opted from an ancestral role in neural ECM binding to a new role in binding SMPs in larval and adult shells in *Crepidula*.

### Are ID proteins responsible for shell molecular self-assembly?

Molluscan shell proteomes frequently contain ID domains, which are regions of a protein that do not undergo conformational folding into a tertiary structure unless bound to ligands, receptors, proteins, or under the right physiological conditions (Uversky 2019). At least 39% of SMPs in *C. fornicata's* shell proteome contain predicted ID regions. Recent computational studies suggest that collections of ID proteins contribute to the molecular self-assembly of the molluscan shell matrix by forming gel microenvironments conducive for mineralization to occur (Pancsa et al. 2019; Marin 2020). It has been speculated that compositionally biased regions in ID SMPs, particularly in aspartic-acid residues (Weiner and Hood 1975), hydrophobic residues like glycine and proline (Marie et al. 2010), or other single residue repeats, may function in binding calcium ions or in creating gel-like microenvironments for crystal precipitation (Marin 2020). Many of the lineage-restricted SMPs in *C. fornicata* contain these compositional biases. In particular, the shell gland restricted genes, CfSMP1 and CfSMP2, have extensive regions of predicted intrinsic disorder: 15% of CfSMP1’s coding sequence, and 40% of CfSMP2’s coding sequence, are predicted to be ID. In molluscs, SMPs with ID regions are thought to have low binding affinity with proteins and polysaccharides, but have greater affinity for inorganic crystals like calcite and aragonite (Marie et al. 2013). If so, then ID SMPs (or supramolecular assemblies thereof) may bind aragonite within the ECM, and contribute to the hierarchical organization of the shell ECM in *C. fornicata*. To test this hypothesis, one approach is to isolate ID proteins, subject them to calcium carbonate *in-vitro* crystallization assays similar to those performed by Rivera-Perez et al. (2020), and elucidate the peptide structure using Nuclear Magnetic Resonance (NMR).

### Components of the *C. fornicata* shell *GRN* and the future of molluscan shell GRNs

Building a shell GRN that explains larval shell gland specification will allow us to understand how biomineralization cell types differentiate (Jackson and Degnan 2016). The 10 SMPs identified in this study are the first-described components of the downstream effector genes of the larval shell GRN in *C. fornicata*. Interestingly, only 2 of the 10 SMPs were located exclusively in the shell gland, while the remaining 8 SMPs were expressed in the shell gland and stomodeum during development. Expression in locations like the stomodeum could have interesting implications for the evolution of SMPs and their function. One explanation is that these effector SMPs may have been co-opted from a stomodeum developmental GRN into a shell gland GRN. Upstream components of a shell gland GRN are still needed to begin to assemble a shell gland GRN. To date, at least 24 transcription factors have been shown to be expressed in the shell gland during embryonic development (Perry et al. 2015; Osborne et al. 2018; Lyons et al. 2020; Truchado-Garcia et al. 2021). The next challenge is determining the epistatic relationships between nodes within the network, which will require knockdowns of transcription factors and effector SMPs like CfSMP1 and CfSMP2. This is perhaps the greatest barrier to building a shell GRN in molluscs: few molluscan models have the ability to perturb the function of genes (Davison and Neiman 2021). Of the few studies that have knocked down SMPs, most have been performed on the bivalve *Pinctada* using RNAi approaches (Jackson and Degnan 2016). *C. fornicata* is now a well-positioned gastropod species for studying biomineralization, due to its established gene perturbation tools (Perry and Henry 2015), and candidate lists of effector SMPs and transcription factors of a shell GRN to perturb. For example, the identification of cell-type specific genes will greatly assist in single-cell sequencing by providing shell gland cellular identity in RNAseq datasets. Moreover, recent advances in computational network theory will allow us to identify additional putative regulators of SMPs (Sleight et al. 2020; Cerveau and Jackson 2021) and help fill the gaps in the shell GRN. Ultimately, a shell GRN will permit the comparison of GRNs between different stages of development, and between different species, to understand the mechanistic underpinnings of molluscan biomineralization.

## Materials and methods

### Sample preparation for electron microscopy and PXRD

Fracture surfaces were prepared by breaking the shell approximately normal to the anterior-posterior axis. For electron microscopy, shell fragments and air-dried sections were mounted on aluminum SEM stubs using double-sided carbon tape and coated with 20 nm of Au/Pd. Samples were imaged using a Hitachi SU8030 SEM equipped with a field emission source. Images were recorded at 2 keV acceleration voltage and a working distance of 8.0 mm, using secondary electron contrast. Three shells from individual *C. fornicata* adults, and approximately 1 g of geological aragonite (Top Minerals, Czech Republic) were ground and powdered separately using a mortar and pestle. The powder samples analyzed under PXRD do not constitute a whole shell, but should be representative of the entire shell since they were mixed well. Powders were analyzed with a voltage of 40 kV and a tube current of 44 mA with a 5 mm slit on a Rigaku Ultima to obtain PXRD data (Supplementary Note 4).

### Shell preparation

Approximately 12–15 adult shells were treated with cold, 6% active sodium hypochlorite solution (Acros Organics) for 2 h, with solution changes every 30 min. Shells were washed with deionized water, allowed to dry, and visually examined for remnants of organic debris. Shells were ground to a fine powder using mortar and pestle followed by addition of a homogenization buffer (4.5 M Guanidine Isothiocyanate, 5% (v/v) β-mercaptoethanol, 0.05 M sodium citrate (pH 7.0), 0.5% (w/v) Sarkosyl) (Flores and Livingston 2017). Minerals were completely dissolved by addition of acetic acid (∼40 mL 25% (v/v)/g mineral). Both precipitate and supernatant were transferred to Spectra/Por 6 Dialysis Membrane MWCO 1000 (Spectrum Laboratories) for dialysis against deionized water. Dialysis was performed at 4°C, with three solution changes over a 12 h period. Following dialysis, the acid soluble matrix found in the supernatant was concentrated using an Amicon Ultra-15 filtration unit followed by an Amicon Ultra-0.5 filter unit.

### Sample preparation for proteomic analysis

Soluble and insoluble fractions were processed by SDS-PAGE using a 10% Bis-Tris NuPAGE gel (Invitrogen) with the MES buffer system, and the gel was run approximately 5 cm. The mobility region was excised into 20 equal sized segments for further processing by in-gel digestion. In-gel digestion was performed on each sample using a robot (ProGest, DigiLab) with the following protocol: (1) Wash with 25 mM aqueous ammonium bicarbonate followed by acetonitrile,  (2) Reduce with 10 mM aqueous dithiothreitol at 60°C followed by alkylation with 50 mM aqueous iodoacetamide at room temperature, (3) Digest each band with 200ng trypsin (Promega) at 37°C for 4 h, (4) quench with formic acid. The resulting supernatant was analyzed directly without further processing.

### Mass spectrometry

Gel digests were sent to MS Bioworks LCC, Ann Arbor MI, for LC-MS/MS. Each gel digest was analyzed by nano LC-MS/MS with a Waters NanoAcquity HPLC system interfaced to a ThermoFisher Q Exactive. Peptides were loaded on a trapping column and eluted over a 75 μm analytical column at 350 nL/min; both columns were packed with Luna C18 resin (Phenomenex). The mass spectrometer was operated in data-dependent mode, with the Orbitrap operating at 60,000 FWHM and 17,500 FWHM for MS and MS/MS, respectively. The 15 most abundant ions were selected for MS/MS.

### RNA collection and sequencing

Adult individuals of *C.fornicata* were collected from Woods Hole, MA, by the Marine Resources Center at the Marine Biological Laboratory. To reduce noise in expression data due to different states of shell mineralization, 30 individuals of *C. fornicata* were grouped into three pools of ten individuals. Mantle, gill, foot, and head were dissected from all 30 individuals. Samples were kept separate and homogenized using mortar and pestle in TRIzol (Life Technologies). Total RNA was extracted according to manufacturer instructions. For each tissue within a pool, one µg of total RNA was collected from all ten individuals and combined to form one replicate, resulting in three replicates for each tissue type. Total RNA was sent to the IGM facility at UCSD for quality and quantity check on an Agilent Tapestation. All samples passed QC with RNA Integrity Numbers (RIN) above 7. RNA was reverse transcribed into cDNA using a TruSeq RNA Sample Prep Kit (Illumina) and paired-end (100 bp) sequenced on a single lane using the HiSeq4000 platform (Illumina).

### Transcriptome assembly and differential expression analysis

Raw reads (325,488,920) were trimmed of adapter sequences and filtered using trimmomatic v0.36 using default settings (Bolger et al. 2014), resulting in 320,960,727 remaining reads: each sample replicate had an average of 26,746,727 trimmed reads. Using these filtered reads from all four tissues and replicates, a multi-tissue transcriptome was *de novo* assembled using Trinity v2.66 with default parameters (Table S6), and was the transcriptome used to perform differential expression (Haas et al. 2013). A second *de novo* transcriptome (mantle transcriptome) was created using only mantle reads (Table S7), and was used to map peptides identified from LC-MS/MS back to the transcriptome. Filtered paired-end reads from all four tissues and three replicates were aligned back to the multi-tissue transcriptome using bowtie2, and transcript abundance estimation was conducted using RSEM resulting in the creation of an abundance count matrix consisting of transcript expression values used for differential expression analysis. Differential gene expression was conducted using the edgeR (Robinson et al. 2010) Bioconductor package for R using default scripts and protocols contained within the Trinity v2.66 utilities folder. Four pairwise comparisons for each of the tissues were performed, and the most differentially expressed transcripts (FDR corrected *P*-value ≤ 0.001; log2FC ≥ 4) were extracted and hierarchically clustered using Perl scripts that are offered through the utilities folder in Trinity v2.66 (Haas et al. 2013).

### Whole mount *in situ* hybridization (WMISH)

Embryos of *C. fornicata* were collected and reared at room temperature, followed by fixation in 3.7% paraformaldehyde in filtered seawater (FSW) for 1 h. After fixation, embryos underwent methanol dehydration and were stored at -20°C. Digoxegenin-labeled riboprobes were made for each SMP gene fragment using a T7 or SP6 MEGAscript kit (Ambion Inc) with DIG-11-UTP (Roche). WMISH was performed according to previously published protocols (Henry, Perry, Fukui, et al. 2010; Perry et al. 2015) (Supplementary Note 4).

## Data availability

The proteomic and shell matrix protein sequence information supporting this article have been uploaded as part of the supplementary data. The 185 SMP nucleotide and protein sequences are also available on GenBank under accession ON512850-ON513034. The RNA sequencing reads used to assemble the multi-tissue and mantle transcriptome have been uploaded to NCBI under BioProject accession PRJNA722737. The Transcriptome Shotgun Assembly (TSA) project for mantle and multi-tissue transcriptomes have been deposited at DDBJ/EMBL/GenBank under accessions GJYT00000000 and GJYS00000000, respectively.

## Supplementary Material

obac023_Supplemental_Files

## References

[bib1] Agbaje OBA , Thomas DE, Dominguez JG, Mclnerney BV, Kosnik MA, Jacob DE. 2019. Biomacromolecules in bivalve shells with crossed lamellar architecture. J Mater Sci 54:4952–69.

[bib2] Aguilera F , McDougall C, Degnan BM. 2014. Evolution of the tyrosinase gene family in bivalve molluscs: independent expansion of the mantle gene repertoire. Acta Biomater 10:3855–65. 24704693 10.1016/j.actbio.2014.03.031

[bib3] Aguilera F , McDougall C, Degnan BM. 2017. Co-Option and De Novo gene evolution underlie molluscan shell diversity. Mol Biol Evol 34:779–92. 28053006 10.1093/molbev/msw294 PMC5400390

[bib4] Arivalagan J , Yarra T, Marie B, Sleight VA, Duvernois-Berthet E, Clark MS, Marie A, Berland S. 2017. Insights from the shell proteome: biomineralization to adaptation. Mol Biol Evol 34:66–77. 27744410 10.1093/molbev/msw219 PMC5854119

[bib5] Bateman A , Coin L, Durbin R, Finn RD, Hollich V, Griffiths-Jones S, Khanna A, Marshall M, Moxon S, Sonnhammer ELL et al. 2004. The Pfam protein families database. Nucleic Acids Res 32:138D–141. 10.1093/nar/gkh121 PMC308855 14681378

[bib6] Blakemore R. 1975. Magnetotactic bacteria. Science 190:377–9. 170679 10.1126/science.170679

[bib7] Boggild OB. 1930. The shell structure of the mollusks. Det Kongelige Danske Videnskabernes Selskabs Skrifter Naturvidenskabelig og Mathematisk Afdeling, Raekke 9:231–326.

[bib8] Bolger AM , Lohse M, Usadel B. 2014. Trimmomatic: a flexible trimmer for Illumina sequence data. Bioinformatics 30:2114–20. 24695404 10.1093/bioinformatics/btu170 PMC4103590

[bib9] Bouasria M , Khadraoui F, Benzaama M-H, Touati K, Chateigner D, Gascoin S, Pralong V, Orberger B, Babouri L, El Mendili Y. 2021. Partial substitution of cement by the association of Ferronickel slags and *Crepidula fornicata* shells. Jour of Build Engin 33:101587.

[bib10] Carini A , Koudelka T, Tholey A, Appel E, Gorb SN, Melzner F, Ramesh K. 2019. Proteomic investigation of the blue mussel larval shell organic matrix. J Struct Biol 208:107385. 31505249 10.1016/j.jsb.2019.09.002

[bib11a] Carter JG , Clark GR. 1985. Classification and phylogenetic significance of molluscan shell microstructure. Series in Geology, Notes for Short Course, 13:50–71.

[bib11] Cerveau N , Jackson DJ. 2021. A survey of miRNAs involved in biomineralization and shell repair in the freshwater gastropod *Lymnaea stagnalis*. Discover Materials 1:7.

[bib12] Dauphin Y , Cuif J-P, Cotte M, Salomé M. 2012. Structure and composition of the boundary zone between aragonitic crossed lamellar and calcitic prism layers in the shell of *Concholepas concholepas* (Mollusca, Gastropoda). Invertebrate Biology 131:165–76.

[bib14a] Davison A , Neiman M. 2021. Mobilizing molluscan models and genomes in biology. Philosophical Transactions of the Royal Society B 376:20200163. 10.1098/rstb.2020.0163 PMC8059959 33813892

[bib13] Delucchi M , Schaper E, Sachenkova O, Elofsson A, Anisimova M. 2020. A new census of protein tandem repeats and their relationship with intrinsic disorder. Genes 11:407. 10.3390/genes11040407 PMC7230257 32283633

[bib14] Dosztányi Z , Csizmok V, Tompa P, Simon I. 2005. IUPred: web server for the prediction of intrinsically unstructured regions of proteins based on estimated energy content. Bioinformatics 21:3433–4. 15955779 10.1093/bioinformatics/bti541

[bib15] Douglas S , Beveridge TJ. 1998. Mineral formation by bacteria in natural microbial communities. FEMS Microbiol Ecol 26:79–88.

[bib16] Dupraz C , Visscher PT. 2005. Microbial lithification in marine stromatolites and hypersaline mats. Trends Microbiol 13:429–38. 16087339 10.1016/j.tim.2005.07.008

[bib17] Dyson HJ , Wright PE. 2005. Intrinsically unstructured proteins and their functions. Nat Rev Mol Cell Biol 6:197–208. 15738986 10.1038/nrm1589

[bib18] Ettensohn CA. 2009. Lessons from a gene regulatory network: echinoderm skeletogenesis provides insights into evolution, plasticity and morphogenesis. Development 136:11–21. 19060330 10.1242/dev.023564

[bib19] Farre B , Dauphin Y. 2009. Lipids from the nacreous and prismatic layers of two *Pteriomorpha* mollusc shells. Comp Biochem Physiol B: Biochem Mol Biol 152:103–9. 18955152 10.1016/j.cbpb.2008.10.003

[bib20] Flores RL , Livingston BT. 2017. The skeletal proteome of the sea star *Patiria miniata* and evolution of biomineralization in echinoderms. BMC Evol Biol 17:125. 28583083 10.1186/s12862-017-0978-z PMC5460417

[bib21] Fukuda I , Ooki S, Fujita T, Murayama E, Nagasawa H, Isa Y, Watanabe T. 2003. Molecular cloning of a cDNA encoding a soluble protein in the coral exoskeleton. Biochem Biophys Res Commun 304:11–7. 12705876 10.1016/s0006-291x(03)00527-8

[bib22] Haas BJ , Papanicolaou A, Yassour M, Grabherr M, Blood PD, Bowden J, Couger MB, Eccles D, Li B, Lieber M et al. 2013. De novo transcript sequence reconstruction from RNA-seq using the trinity platform for reference generation and analysis. Nat Protoc 8:1494–512. 23845962 10.1038/nprot.2013.084 PMC3875132

[bib23] Hejnol A , Martindale MQ, Henry JQ. 2007. High-resolution fate map of the snail *Crepidula fornicata*: the origins of ciliary bands, nervous system, and muscular elements. Dev Biol 305:63–76. 17346693 10.1016/j.ydbio.2007.01.044

[bib24] Henry JJ , Collin R, Perry KJ. 2010. The slipper snail, *Crepidula*: an emerging lophotrochozoan model system. Biol Bull 218:211–29. 20570845 10.1086/BBLv218n3p211

[bib25] Henry JJ , Perry KJ, Fukui L, Alvi N. 2010. Differential localization of mRNAs during early development in the mollusc, *Crepidula fornicata*. Integr Comp Biol 50:720–33. 21558235 10.1093/icb/icq088

[bib26] Henry JQ , Lyons DC. 2016. Molluscan models: *Crepidula fornicata*. Current Opinions in Genet & Developmental Biology 39:138–48. 10.1016/j.gde.2016.05.021 27526387

[bib27] Henry JQ , Perry KJ, Martindale MQ. 2010. β-catenin and early development in the gastropod, *Crepidula fornicata*. Integr Comp Biol 50:707–19. 21558234 10.1093/icb/icq076

[bib28a] Hirotsune S , Takahara T, Sasaki N, Hirose K, Yoshiki A, Ohashi T, Kusakabe M, Murakami Y, Muramatsu M, Watanabe S et al. 1995. The reeler gene encodes a protein with an EGF–like motif expressed by pioneer neurons. Nature genetics 10:77–83. 7647795 10.1038/ng0595-77

[bib28] Hu Y-Y , Rawal A, Schmidt-Rohr K. 2010. Strongly bound citrate stabilizes the apatite nanocrystals in bone. Proc Natl Acad Sci 107:22425–9. 21127269 10.1073/pnas.1009219107 PMC3012505

[bib29] Jackson DJ , Degnan BM. 2016. The importance of evo-devo to an integrated understanding of molluscan biomineralization. J Struct Biol 196:67–74. 26792641 10.1016/j.jsb.2016.01.005

[bib30] Khalturin K , Hemmrich G, Fraune S, Augustin R, Bosch TCG. 2009. More than just orphans: are taxonomically-restricted genes important in evolution? Trends Genet 25:404–13. 19716618 10.1016/j.tig.2009.07.006

[bib31] Knoll AH. 2003. Biomineralization and evolutionary history. Rev Mineral Geochem 54:329–56.

[bib32] Kocot KM , Aguilera F, McDougall C, Jackson DJ, Degnan BM. 2016. Sea shell diversity and rapidly evolving secretomes: insights into the evolution of biomineralization. Frontiers in Zoology 13:23. 27279892 10.1186/s12983-016-0155-z PMC4897951

[bib33] Leão P , Le Nagard L, Yuan H, Cypriano J, Da Silva-Neto I, Bazylinski DA, Acosta-Avalos D, de Barros HL, Hitchcock AP, Lins U et al. 2020. Magnetosome magnetite biomineralization in a flagellated protist: evidence for an early evolutionary origin for magnetoreception in eukaryotes. Environ Microbiol 22:1495–506. 31188524 10.1111/1462-2920.14711

[bib34] Le Roy N , Jackson DJ, Marie B, Ramos-Silva P, Marin F. (2014). The evolution of metazoan α-carbonic anhydrases and their roles in calcium carbonate biomineralization. Frontiers in Zoology 11:1–16.24401080

[bib35] Liu F , Shah DS, Gadd GM. 2021. Role of protein in fungal biomineralization of copper carbonate nanoparticles. Curr Biol 31:358–368.e3. 33176131 10.1016/j.cub.2020.10.044

[bib36] Li XW , Ji HM, Yang W, Zhang GP, Chen DL. 2017. Mechanical properties of crossed-lamellar structures in biological shells: a review. J Mech Behav Biomed Mater 74:54–71. 28550764 10.1016/j.jmbbm.2017.05.022

[bib37] Lowenstam HA , Weiner S. 1989. On biomineralization. Oxford, UK: Oxford University Press. on Demand.

[bib40a] Lyons DC , Henry JQ. 2022. Slipper snail tales: How Crepidula fornicata and Crepidula atrasolea became model molluscs. Curr Top Dev Biol 147:375–99. 35337456 10.1016/bs.ctdb.2021.12.013 PMC9187910

[bib38] Lyons DC , Perry KJ, Batzel G, Henry JQ. 2020. BMP signaling plays a role in anterior-neural/head development, but not organizer activity, in the gastropod *Crepidula fornicata*. Dev Biol 463:135–57. 32389712 10.1016/j.ydbio.2020.04.008 PMC7444637

[bib39] Lyons DC , Perry KJ, Henry JQ. 2015. Spiralian gastrulation: germ layer formation, morphogenesis, and fate of the blastopore in the slipper snail *Crepidula fornicata*. Evodevo 6:24. 26664718 10.1186/s13227-015-0019-1 PMC4673862

[bib40] Lyons DC , Perry KJ, Henry JQ. 2017. Morphogenesis along the animal-vegetal axis: fates of primary quartet micromere daughters in the gastropod *Crepidula fornicata*. BMC Evol Biol 17:217. 28915788 10.1186/s12862-017-1057-1 PMC5603038

[bib41] Lyons DC , Perry KJ, Lesoway MP, Henry JQ. 2012. Cleavage pattern and fate map of the mesentoblast, 4d, in the gastropod *Crepidula*: a hallmark of spiralian development. Evodevo 3:21. 22992254 10.1186/2041-9139-3-21 PMC3724503

[bib42] Mann K , Edsinger E. 2014. The *Lottia gigantea* shell matrix proteome: re-analysis including MaxQuant iBAQ quantitation and phosphoproteome analysis. Proteome Sci 12:28. 25018669 10.1186/1477-5956-12-28 PMC4094399

[bib43] Mann K , Edsinger-Gonzales E, Mann M. 2012. In-depth proteomic analysis of a mollusc shell: acid-soluble and acid-insoluble matrix of the limpet *Lottia gigantea*. Proteome Sci 10:28. 22540284 10.1186/1477-5956-10-28 PMC3374290

[bib44] Mann S. (1983). Mineralization in biological systems. Inorganic elements in biochemistry (pp. 125–74). Springer, Berlin, Heidelberg.

[bib45] Marie B , Jackson DJ, Ramos-Silva P, Zanella-Cléon I, Guichard N, Marin F. 2013. The shell-forming proteome of *Lottia gigantea* reveals both deep conservations and lineage-specific novelties. FEBS J 280:214–32. 23145877 10.1111/febs.12062

[bib46] Marie B , Marie A, Jackson DJ, Dubost L, Degnan BM, Milet C, Marin F. 2010. Proteomic analysis of the organic matrix of the abalone *Haliotis asinina* calcified shell. Proteome Sci 8:54. 21050442 10.1186/1477-5956-8-54 PMC2989941

[bib47] Marin F. 2020. Mollusc shellomes: past, present, and future. J Struct Biol 212:107583. 32721585 10.1016/j.jsb.2020.107583

[bib48] Marin F , Luquet G. 2004. Molluscan shell proteins. CR Palevol 3:469–92.

[bib49] Marxen JC , Hammer M, Gehrke T, Becker W. 1998. Carbohydrates of the organic shell matrix and the shell-forming tissue of the snail *Biomphalaria glabrata* (Say). Biol Bull 194:231–40. 28570847 10.2307/1543052

[bib50] McDougall C , Aguilera F, Degnan BM. 2013. Rapid evolution of pearl oyster shell matrix proteins with repetitive, low-complexity domains. J R Soc, Interface 10:20130041. 23427100 10.1098/rsif.2013.0041 PMC3627088

[bib51] McDougall C , Degnan BM. 2018. The evolution of mollusc shells. WIREs Developmental Biology 7:e313. 10.1002/wdev.313 29470863

[bib52] Newman AM , Cooper JB. 2007. XSTREAM: a practical algorithm for identification and architecture modeling of tandem repeats in protein sequences. BMC Bioinf 8:1–19. 10.1186/1471-2105-8-382 PMC2233649 17931424

[bib53] Noll F , Sumper M, Hampp N. 2002. Nanostructure of diatom silica surfaces and of biomimetic analogues. Nano Lett 2:91–5.

[bib54] Osborne CC , Perry KJ, Shankland M. 2018. Ectomesoderm and epithelial–mesenchymal transition related genes in spiralian development. Dev Dyn 247:1097–120. 30133032 10.1002/dvdy.24667

[bib55] Pancsa R , Schad E, Tantos A, Tompa P. 2019. Emergent functions of proteins in non-stoichiometric supramolecular assemblies. Biochimica et Biophysica Acta (BBA)—Proteins and Proteomics 1867:970–9. 30826453 10.1016/j.bbapap.2019.02.007

[bib56] Perry KJ , Henry JQ. 2015. CRISPR/C as9-mediated genome modification in the mollusc, *Crepidula fornicata*. Genesis 53:237–44. 25529990 10.1002/dvg.22843

[bib57] Perry KJ , Lyons DC, Truchado-Garcia M, Fischer AHL, Helfrich LW, Johansson KB, Diamond JC, Grande C, Henry JQ. 2015. Deployment of regulatory genes during gastrulation and germ layer specification in a model spiralian mollusc *Crepidula*. Dev Dyn 244:1215–48. 26197970 10.1002/dvdy.24308

[bib58] Ponder WF , Colgan DJ, Healy JM, Nutzel A, Strong EE. 2008. Caenogastropoda. In: Ponder WF, Lindberg DR, editors. Phylogeny and evolution of the Mollusca. Berkeley: University of California Press. p. 331–83.

[bib59] Riding R. 1991. Calcified cyanobacteria. In: Riding R, editor. Calcareous Algae and Stromatolites Berlin, Heidelberg: Springer Berlin Heidelberg. p. 55–87.

[bib60] Rivera-Perez C , Flores-Sánchez IA, Ojeda Ramírez de Areyano JJ, Rojas Posadas DI, Hernández-Saavedra NY. 2020. A shell matrix protein of *Pinctada mazatlanica* produces nacre platelets in vitro. Sci Rep 10:20201. 33214608 10.1038/s41598-020-77320-7 PMC7677314

[bib61] Robinson MD , McCarthy DJ, Smyth GK. 2010. edgeR: a Bioconductor package for differential expression analysis of digital gene expression data. Bioinformatics 26:139–40. 19910308 10.1093/bioinformatics/btp616 PMC2796818

[bib62] Schopf JW , Packer BM. 1987. Early Archean (3.3-billion to 3.5-billion-year-old) microfossils from Warrawoona Group, Australia. Science 237:70–3. 11539686 10.1126/science.11539686

[bib63] Sleight VA , Antczak P, Falciani F, Clark MS. 2020. Computationally predicted gene regulatory networks in molluscan biomineralization identify extracellular matrix production and ion transportation pathways. Bioinformatics 36:1326–32. 31617561 10.1093/bioinformatics/btz754 PMC7703775

[bib64] Stegbauer L , Smeets PJM, Free R, Wallace SG, Hersam MC, Alp EE, Joester D. 2021. Persistent polyamorphism in the chiton tooth: from a new biomineral to inks for additive manufacturing. Proc Natl Acad Sci 118:e2020160118. 34088834 10.1073/pnas.2020160118 PMC8202020

[bib65] Suzuki M , Saruwatari K, Kogure T, Yamamoto Y, Nishimura T, Kato T, Nagasawa H. 2009. An acidic matrix protein, Pif, is a key macromolecule for nacre formation. Science 325:1388–90. 19679771 10.1126/science.1173793

[bib66] Truchado-Garcia M , Caccavale F, Grande C, D'Aniello S. 2021. Expression pattern of Nitric Oxide synthase during development of the marine gastropod mollusc, *Crepidula fornicata*. Genes 12:314. 33671839 10.3390/genes12020314 PMC7926364

[bib67] Uversky VN. 2019. Protein intrinsic disorder and structure-function continuum. Prog Mol Biol Transl Sci 166:1–17. 31521229 10.1016/bs.pmbts.2019.05.003

[bib68] Van Der Lee R , Buljan M, Lang B, Weatheritt RJ, Daughdrill GW, Dunker AK, Fuxreiter M, Gough J, Gsponer J, Jones DT et al. 2014. Classification of intrinsically disordered regions and proteins. Chem Rev 114:6589–631. 24773235 10.1021/cr400525m PMC4095912

[bib69] Varney RM , Speiser DI, McDougall C, Degnan BM, Kocot KM. 2021. The iron-responsive genome of the chiton *Acanthopleura granulata*. Genome Biology and Evolution 13:evaa263. 33320175 10.1093/gbe/evaa263 PMC7850002

[bib70] Weiner S , Hood L. 1975. Soluble protein of the organic matrix of mollusk shells: a potential template for shell formation. Science 190:987–9. 1188379 10.1126/science.1188379

[bib71] Wilmot NV , Barber DJ, Taylor JD, Graham AL. 1992. Electron microscopy of molluscan crossed-lamellar microstructure. Philosophical Transactions of the Royal Society London. Series B: Biological Sciences 337:21–35.

[bib72] Wilt FH. 2005. Developmental biology meets materials science: morphogenesis of biomineralized structures. Dev Biol 280:15–25. 15766744 10.1016/j.ydbio.2005.01.019

[bib73] Zhao R , Takeuchi T, Luo Y-J, Ishikawa A, Kobayashi T, Koyanagi R, Villar-Briones A, Yamada L, Sawada H, Iwanaga S, Nagai K et al. 2018. Dual gene repertoires for larval and adult shells reveal molecules essential for molluscan shell formation. Mol Biol Evol 35:2751–61. 30169718 10.1093/molbev/msy172 PMC6231486

