# Supplementary material for: Proteomic and Transcriptomic Analyses in the Slipper Snail Crepidulafornicata Uncover Shell Matrix Genes Expressed During Adult and Larval Biomineralization
Source: Integr Org Biol. 2022 Aug 10;4(1):obac023. doi: 10.1093/iob/obac023 (PMC9365450; doi:10.1093/iob/obac023)
Supplement: obac023_Supplemental_Files [file obac023_supplemental_files.zip › CLEAN_IOB_Revision_Supplemental_Figures_Tables.pdf]

Supplementary Figures

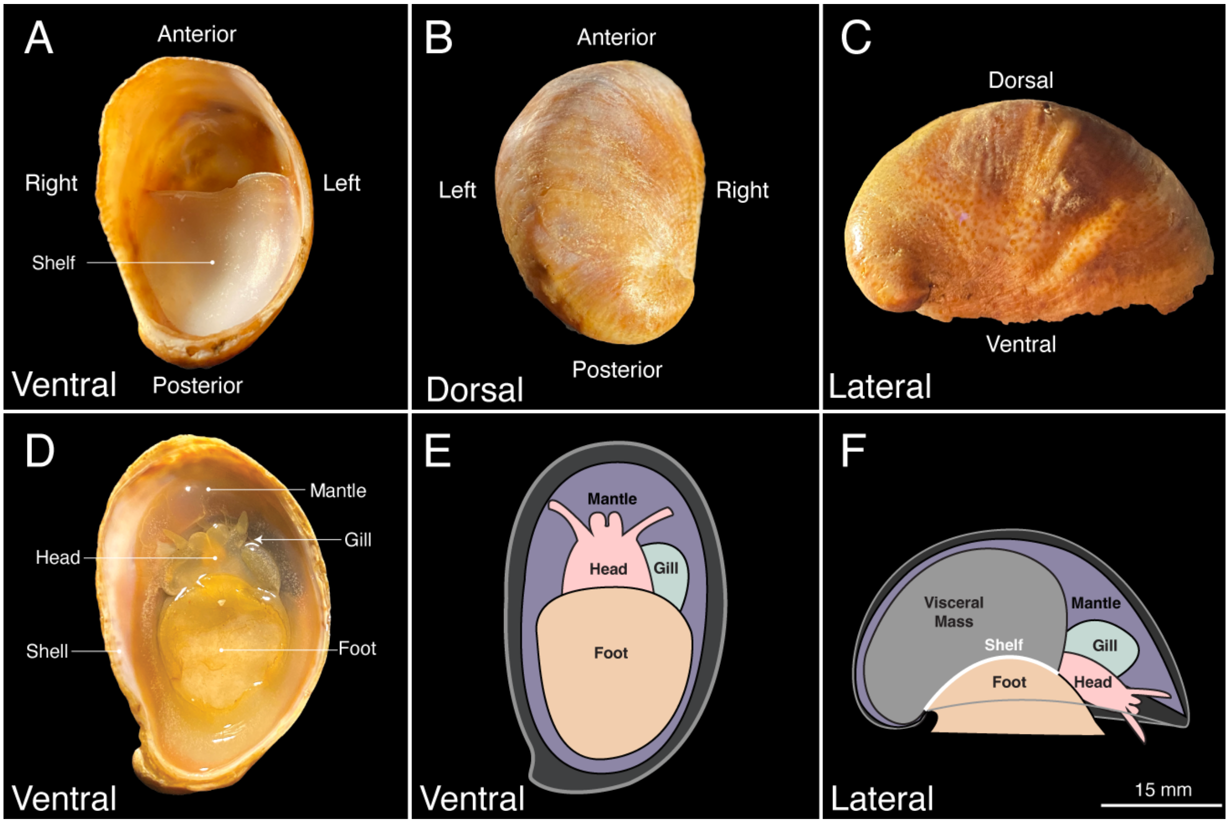

Figure S1: Anatomy and shell morphology of an adult specimen of *C. fornicata*. A: Ventral view of an adult shell. Note the presence of a slightly convex "shelf" that attaches to both sides of the ventral shell. **B:** Dorsal view of an adult shell. **C:** Lateral view of an adult shell. **D:** Ventral view of a living adult specimen. Note the exposed foot, mantle, and head organs. The gill is located behind the head. **E-F:** Diagram depicting the anatomy of *C. fornicata*. Note: In panel F, the shelf sits between the foot and visceral mass of the animal.

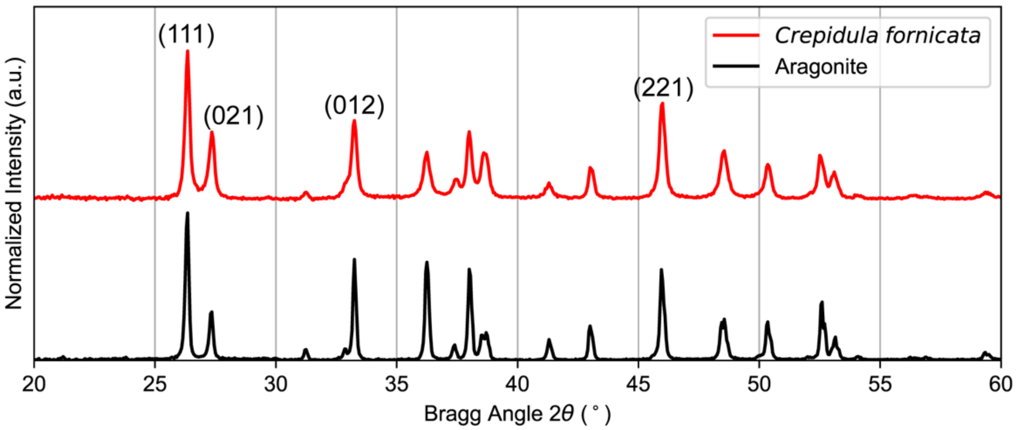

Figure S2: Powder X-ray diffraction (PXRD) patterns of *C. fornicata* shell and geological aragonite reference standard. Plot of diffracted intensity, normalized to that of the (111) reflection, against the Bragg angle.

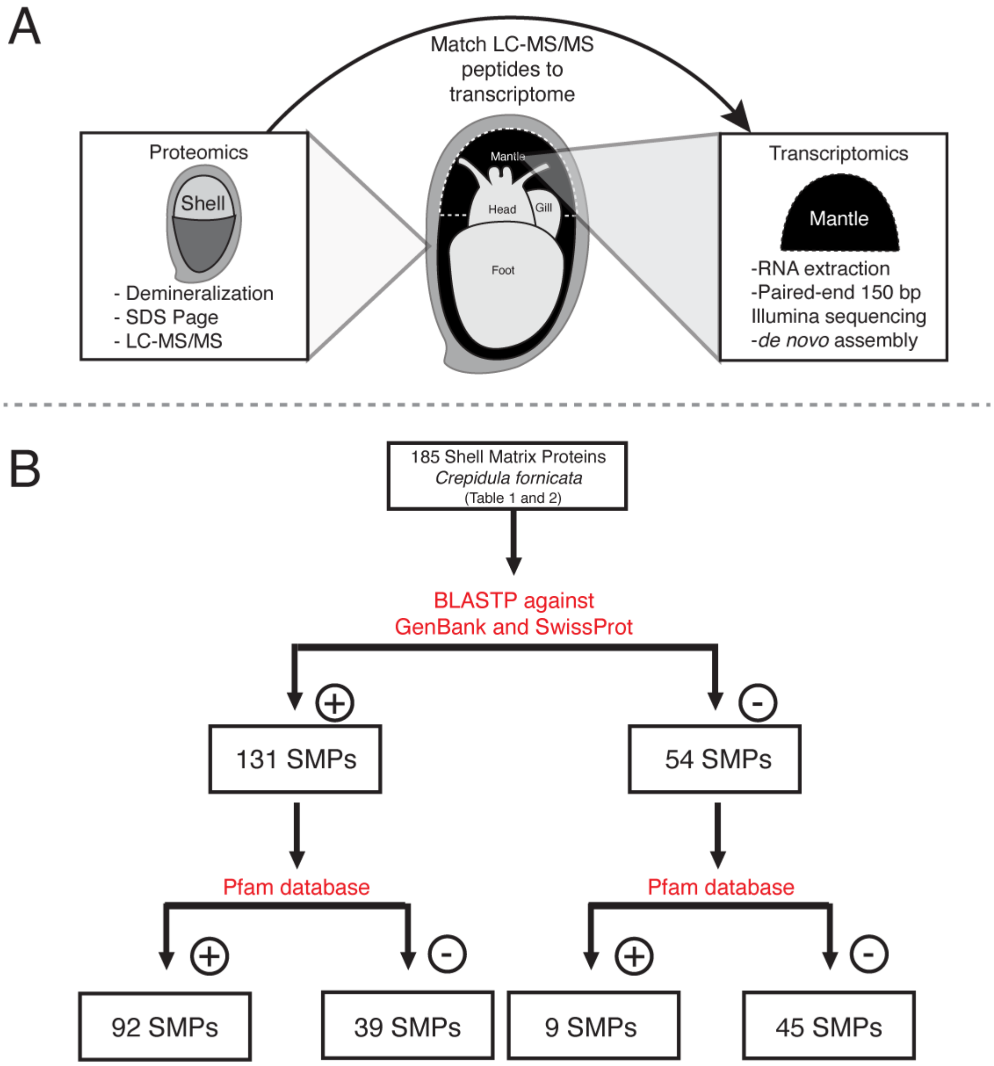

Figure S3: Methods for identifying and annotating shell matrix proteins in *C. fornicata*. **A:** Proteomic and transcriptomic methods for identifying shell matrix proteins (SMPs) in *C. fornicata*. SMPs were extracted from shells, digested, and peptides were analyzed by MS/MS and mapped to a mantle tissue transcriptome. **B:** Annotation pipeline for all 185 SMPs. SMPs were annotated by BLASTP searches against the Genbank database. Conserved domains were identified by HMMER searches against the Pfam database. Plus and minus sign above each box indicates SMPs that have or don't have matches to Genbank or Pfam.

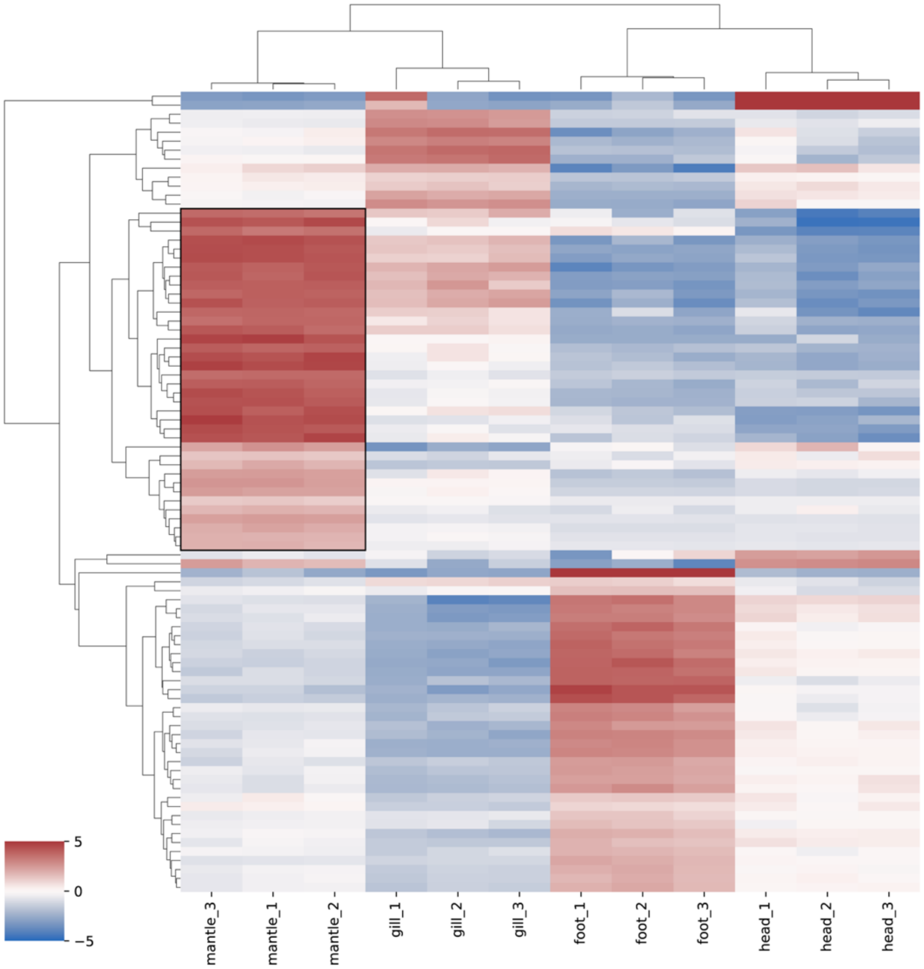

Figure S4: Heatmap of differentially expressed genes. Differential expression was determined using a p-value of <0.001 and a fold-change >4. Tissue samples and their biological replicates are represented in columns; differentially expressed genes are depicted as horizontal lines (rows). Genes are hierarchically clustered (y-axis) based on gene expression profiles, with more similar genes clustering together. Over expressed genes are colored red, while under expressed genes are colored blue. The Black box in the mantle column indicate a cluster of differentially expressed genes expressed in the mantle compared to the head, foot and gill.

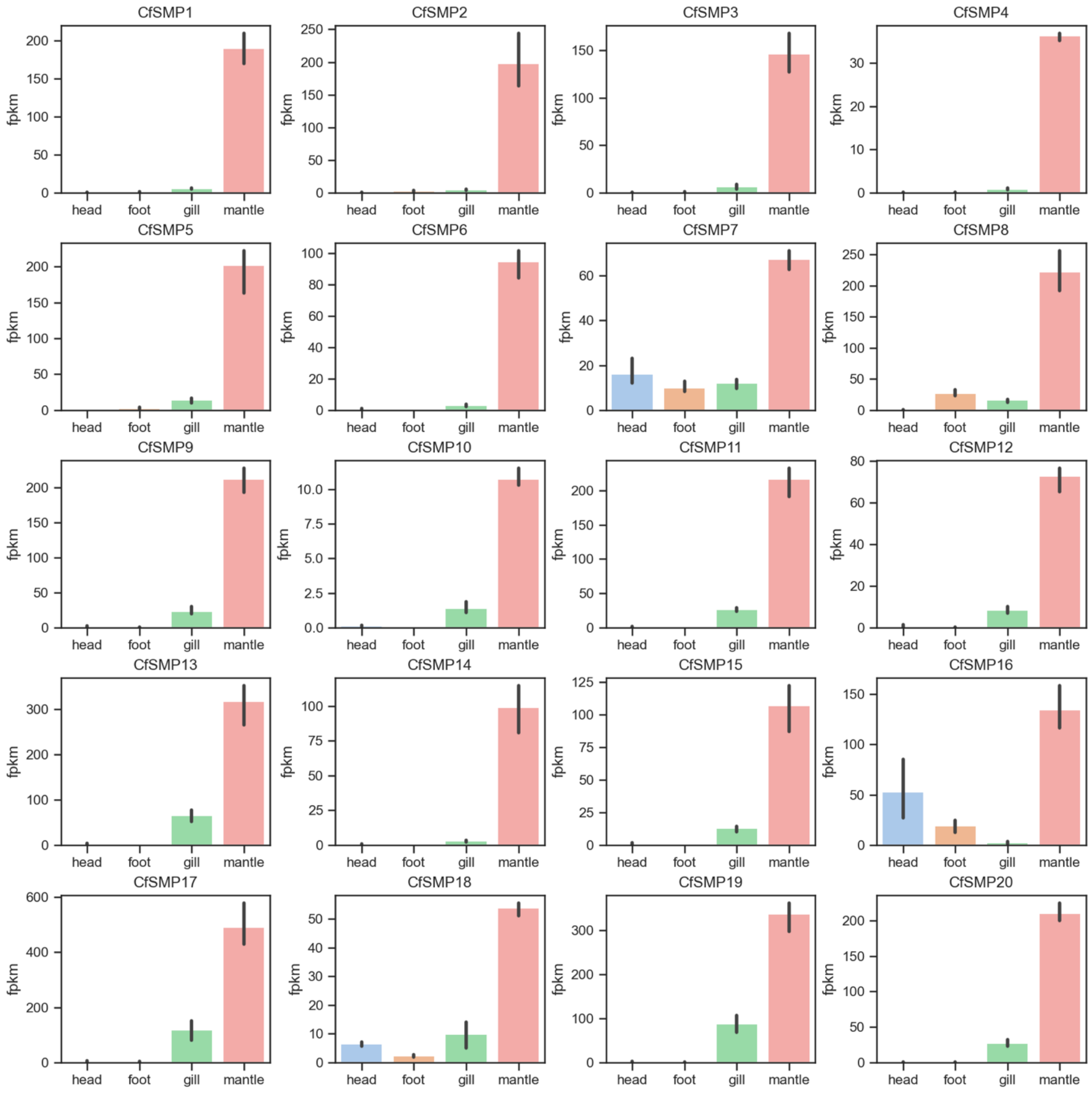

Figure S5: Expression levels of 20 differentially expressed shell matrix proteins in four adult tissues of *C. fornicata*. Normalized-FPKM average expression for 20 SMPs. All SMPs are significantly differentially expressed in the mantle compared to the foot, gill, and head (FDR corrected P-value < 0.001).

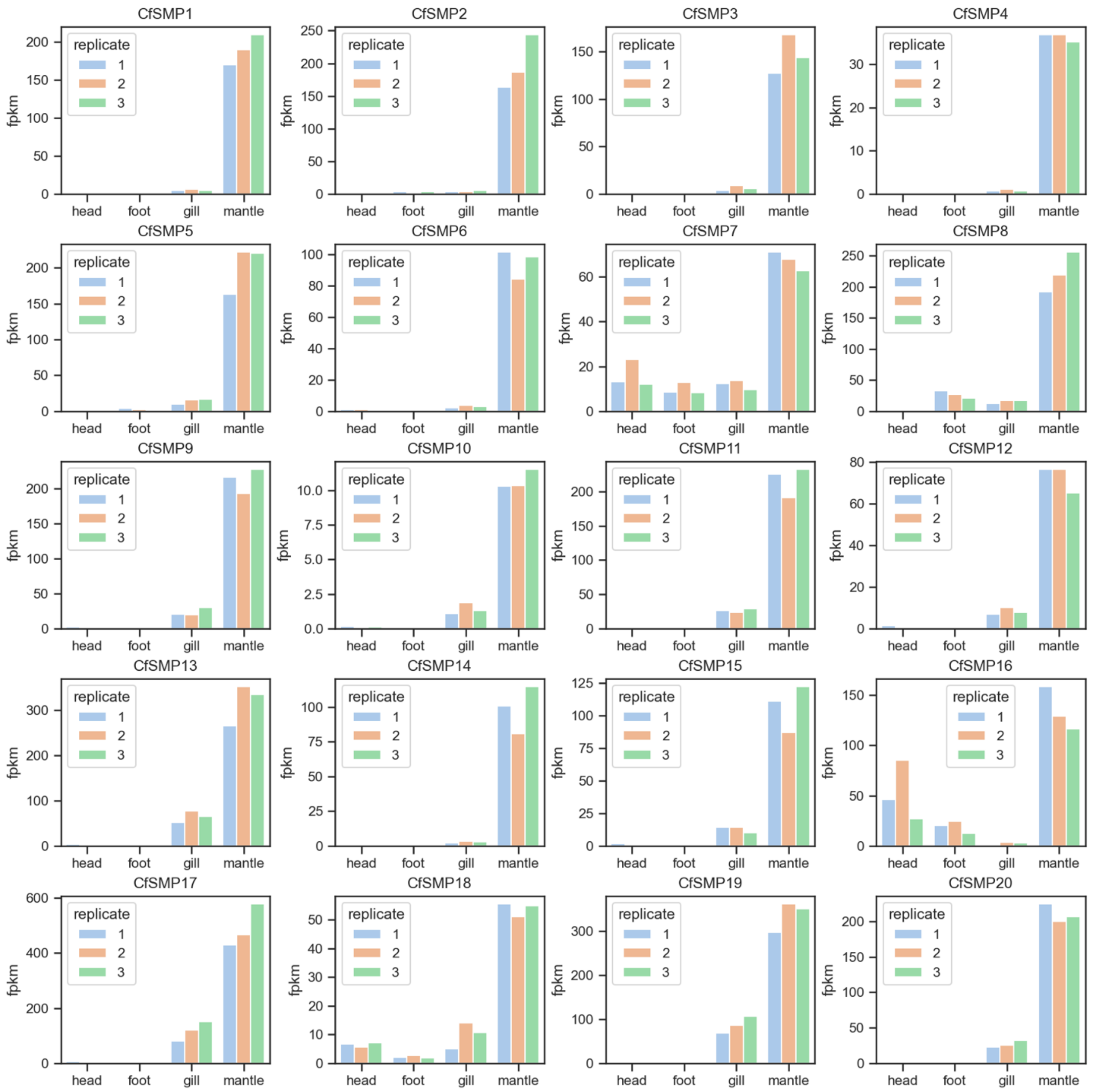

Figure S6: Expression levels for individual replicates of all 20 differentially expressed shell matrix proteins in four adult tissues of *C. fornicata*. Normalized-FPKM expression for 20 SMPs. All SMPs are significantly differentially expressed in the mantle compared to the foot, gill, and head (FDR corrected P-value < 0.001).

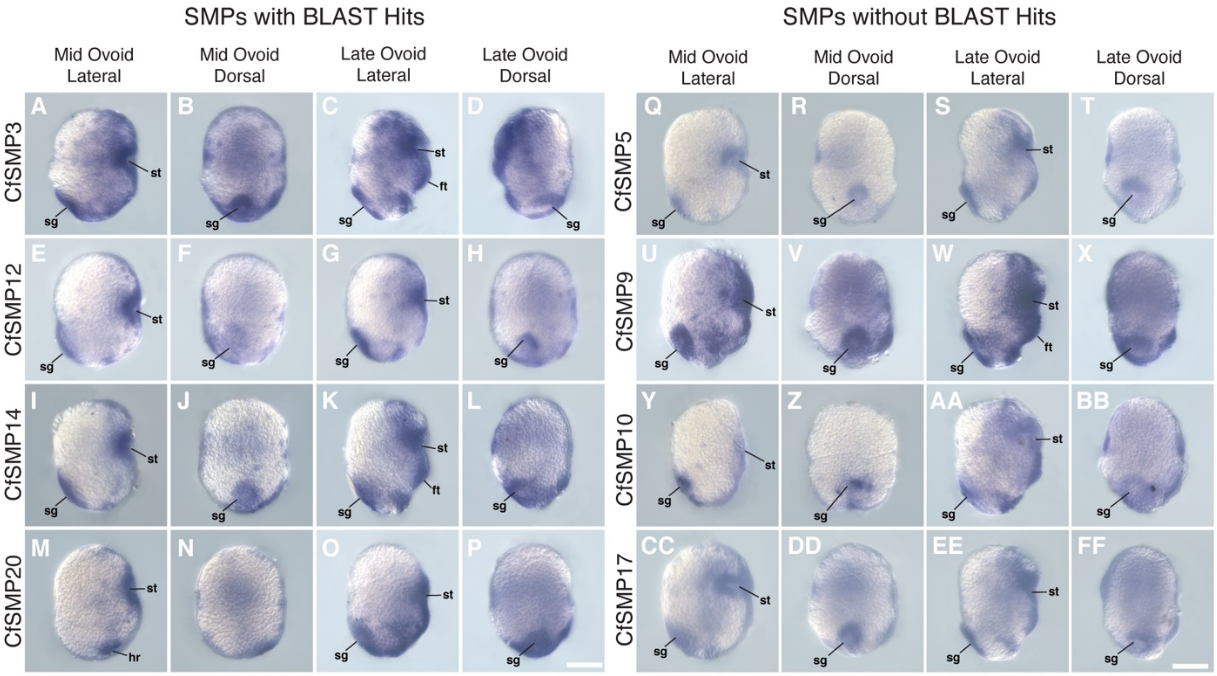

Figure S7: Expression of 8 SMPs during larval development in *C. fornicata*. Eight differentially expressed SMPs were screened during shell gland development. **A-P:** Expression of 4 differentially expressed SMPs that have BLAST hits. **Q-FF:** Expression of 4 differentially expressed SMPs without BLAST hits. All specimens oriented as right lateral or dorsal views, with anterior up and posterior down. Detailed descriptions for each gene can be found in the Results and Supplemental Figures 5-14. Embryo stages are for mid ovoid (120-137 hpf) and late ovoid (140-170 hpf). Structure labels: st, stomodeum; sg, shell gland; vr, velar rudiment Structure labels: st, stomodeum; hr, hindgut rudiment; sg, shell gland; sge, shell gland edge; vr, velar rudiment; vl, velar lobe; oc, ocelli; ft, fo;lot. Scale bar in FF = 50 µm.

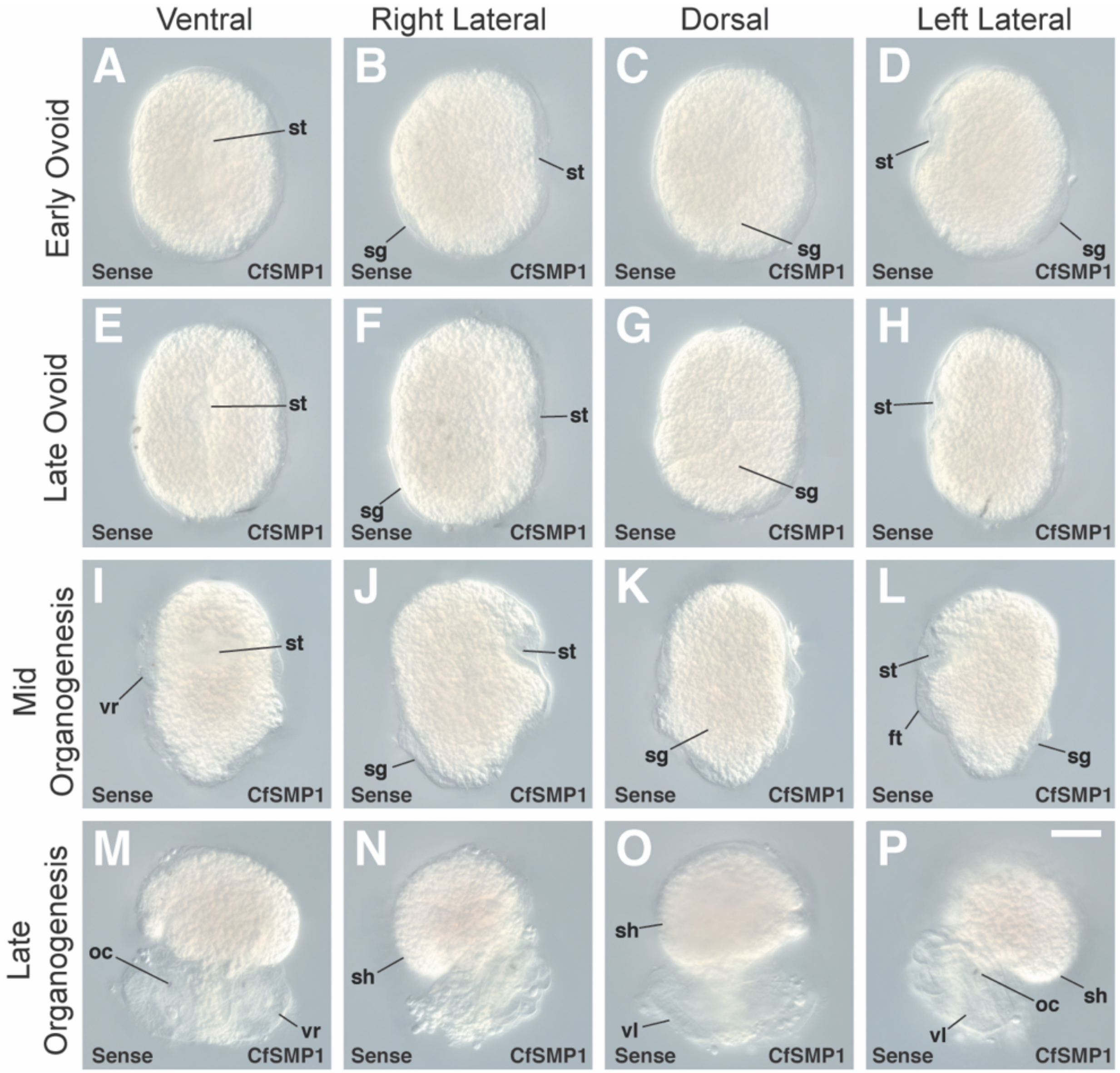

Figure S8: Expression of CfSMP1 Sense during embryogenesis in *C. fornicata*. A-D: Early ovoid (~137 hpf) staged embryos. **E-H:** Late ovoid embryos (~150 hpf). **I-L:** Mid organogenesis embryos (~180 hpf). **M-P:** Late organogenesis staged embryos (~196 hpf).

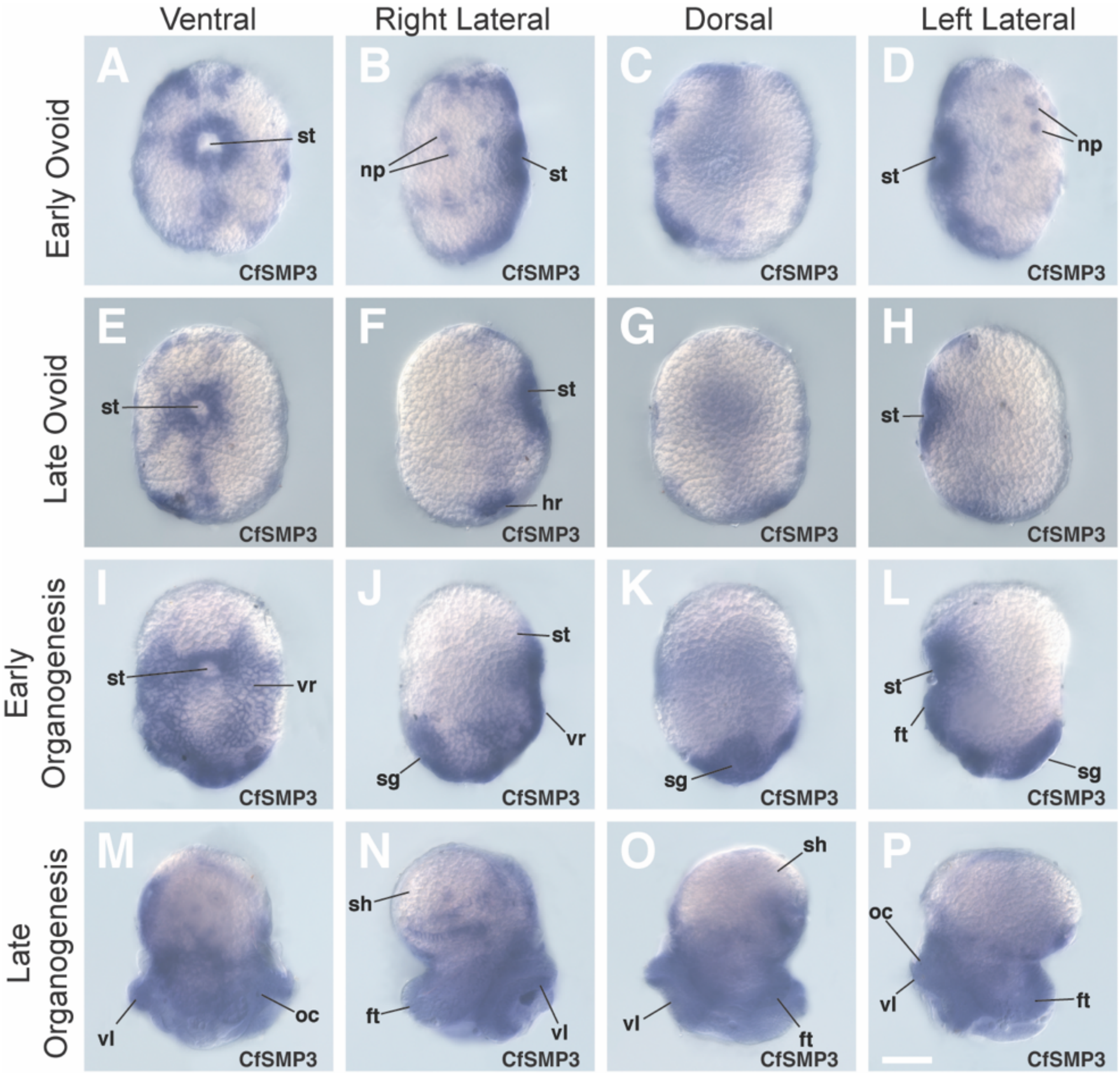

Figure S9: Expression of CfSMP3 during embryogenesis in *C. fornicata.* A-D: CfSMP3 is expressed in the stomodeum in early ovoid staged embryos (~130 hpf). **E-H:** Shell gland expression is apparent at ~150 hpf in mid ovoid staged embryos. Expression is also apparent in the stomodeum and in neural precursor cells on the dorsum. **I-L:** During early organogenesis (~170 hpf), CfSMP3 is expressed in the hindgut, stomodeum, shell gland, and velar rudiments. **M-P:** By veliger stage (~196 hpf), expression is detected in the head, as well as ubiquitously in the shell.

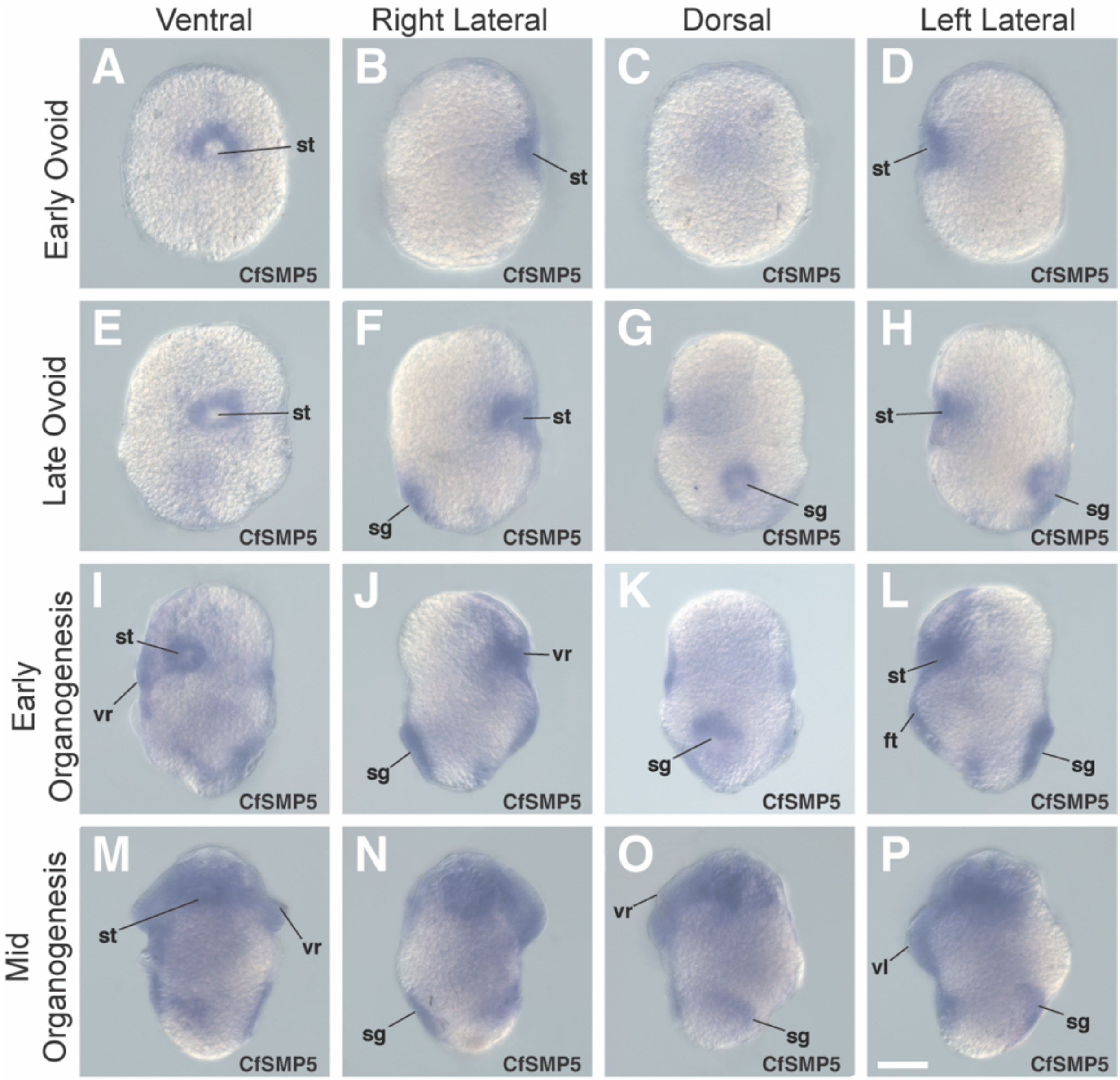

Figure S10: Expression of CfSMP5 during embryogenesis in *C. fornicata.* A-D: CfSMP5 is expressed in the stomodeum in early ovoid embryos (~135 hpf). **E-H:** During late ovoid stages (~150 hpf), CfSMP5 is expressed in the shell gland as well as the stomodeum.**I-L:** By early organogenesis (~170 hpf), expression is present in the velar rudiment, stomodeum, and shell gland. **M-P:** By mid organogenesis (180 hpf), CfSMP5 is expressed in the stomodeum, velar rudiments, and shell gland.

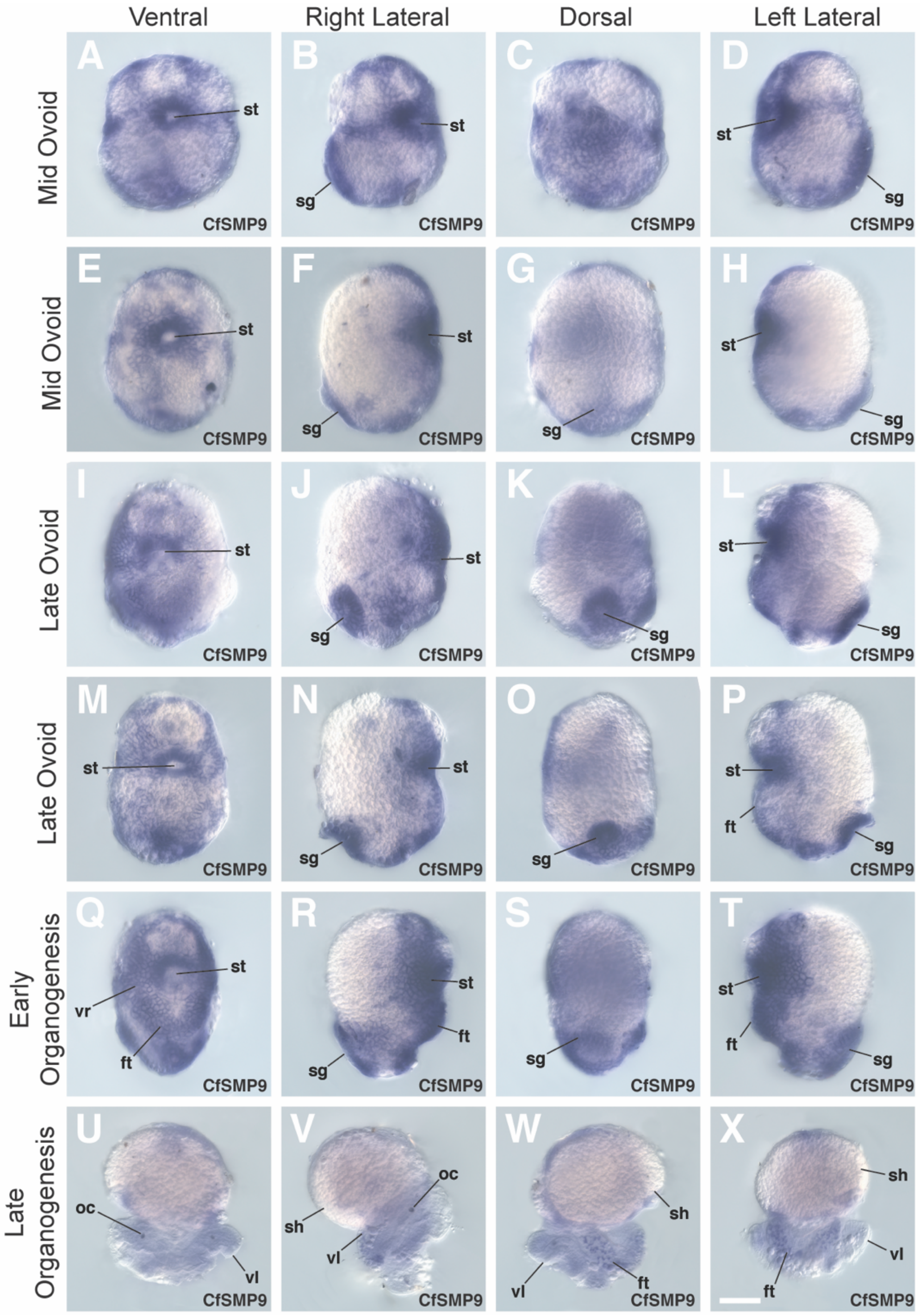

Figure S11: Expression of CfSMP9 during embryogenesis in *C. fornicata.* **A-D:** CfSMP9 is expressed in the stomodeum and on the dorsal surface in early ovoid embryos (137 hpf). **E-H:** Expression persists in the stomodeum and in tissue located around the opening of the stomodeum. Faint shell gland expression is seen at approximately 140 hpf. **I-L:** In late ovoid staged embryos (158 hpf), CfSMP9 is expressed in anteriorly located cells of the stomodeum, and in the shell gland. **M-P:** Shell gland and stomodeum expression persists approximately 2 hours later (160 hpf) in late ovoid embryos. **Q-T:** During early organogenesis (~170 hpf), CfSMP9 is expressed ubiquitously in ventral ectodermal cells, primarily in the foot and velar rudiments, as well as in the stomodeum. Shell gland expression was also detected on the posteodorsal ectoderm.

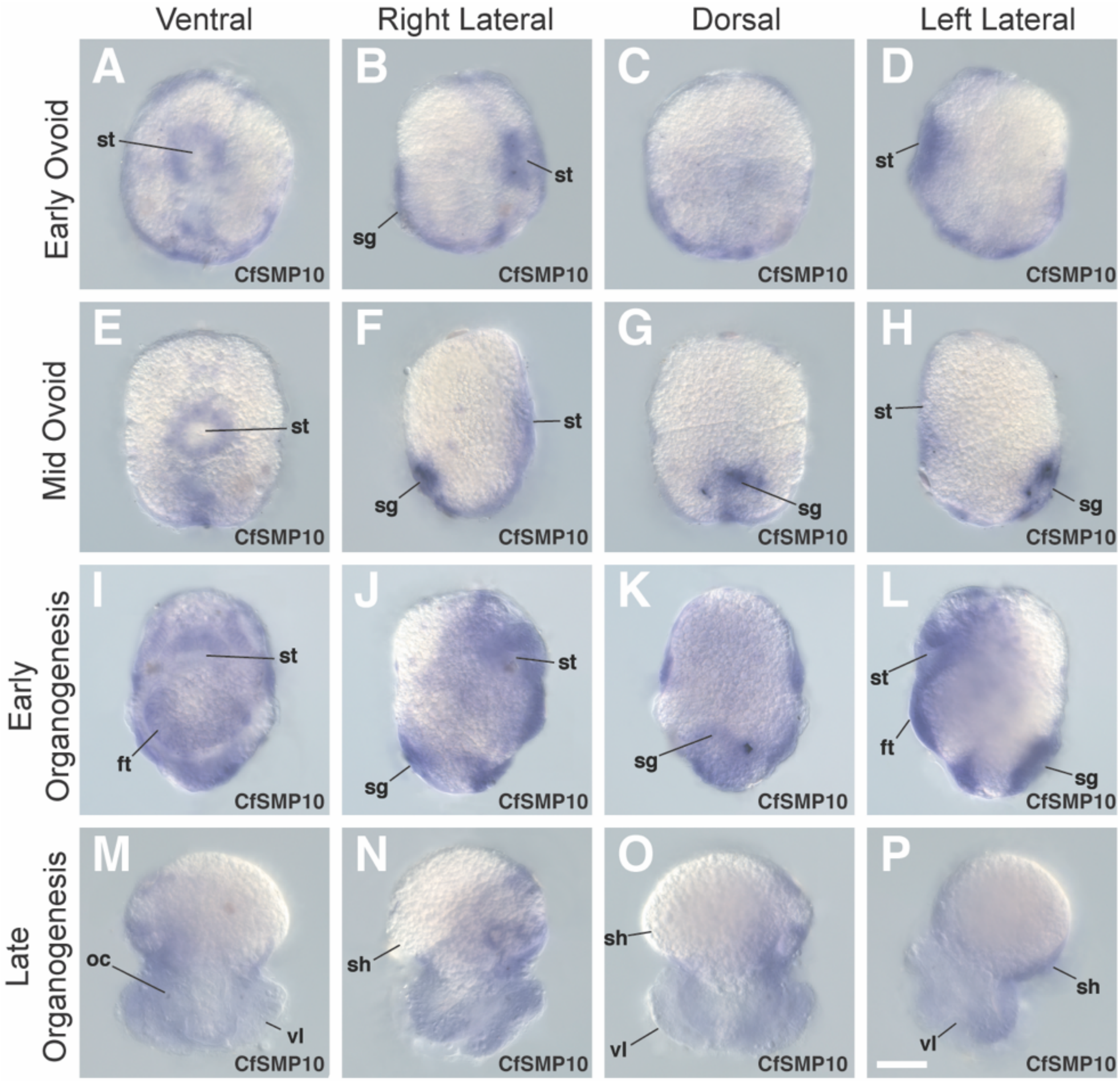

Figure S12: Expression of CfSMP10 during embryogenesis in *C. fornicata.* A-D: In early ovoid embryos (~128 hpf) CfSMP10 was expressed in cells lining the stomodeum. **E-H:** Shell gland expression of CfSMP10 was detected at ~137 hpf. Expression in the stomodeum was also apparent. **I-L:** By early organogenesis (~170 hpf), CfSMP10 is expressed in the stomodeum and shell gland, as well as in the foot rudiment. **M-P:** In veliger larvae (~196 hpf), CfSMP10 was detected in the foot, velar lobes, and in the presumptive kidney.

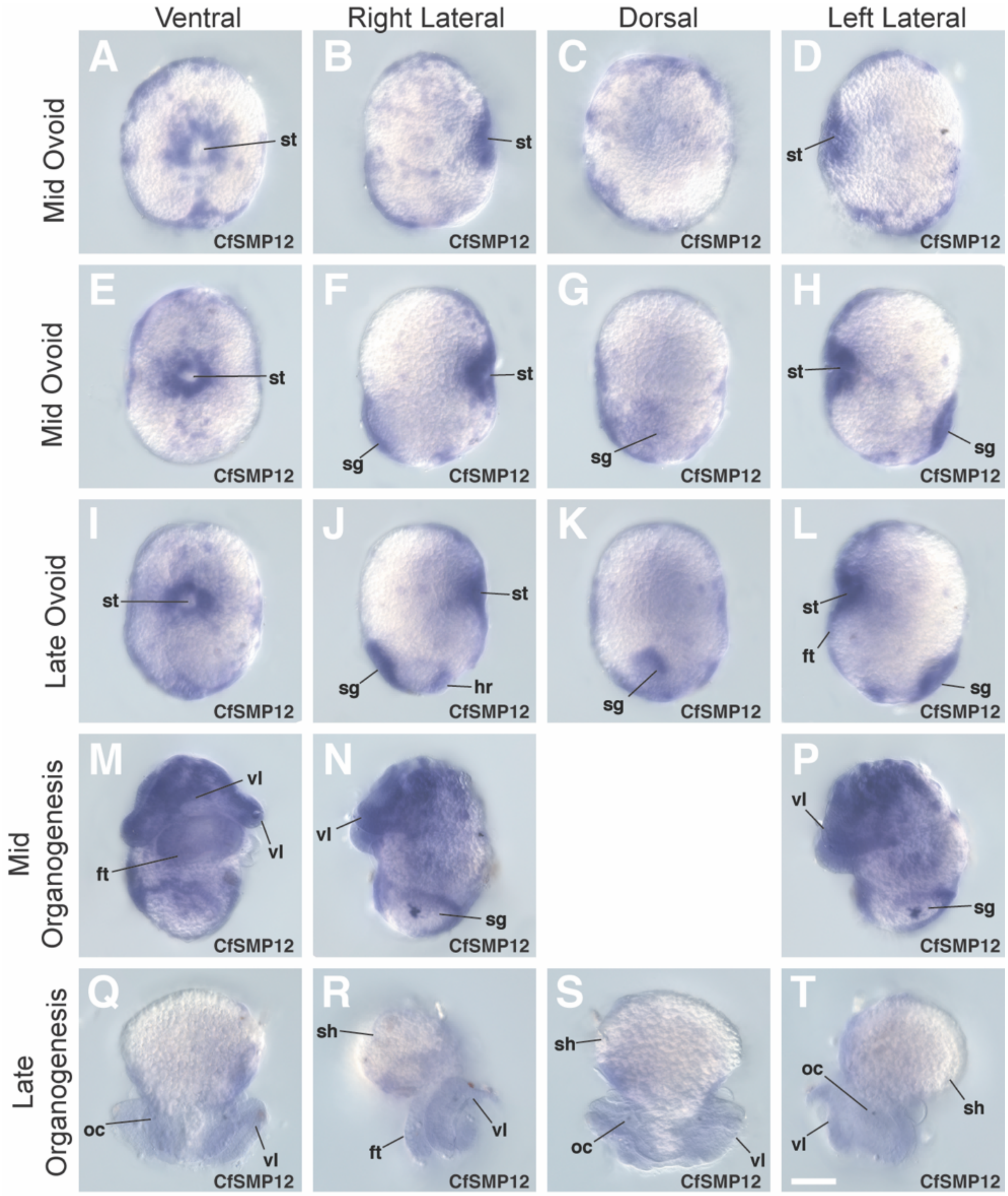

Figure S13: Expression of CfSMP12 during embryogenesis in *C. fornicata.* A-D: Expression of CfSMP12 was detected around the stomodeum in mid ovoid stages (135 hpf). **E-H:** Approximately five hours later (~140 hpf) faint shell gland expression was detected. **I-L:** Expression in stomodeum and shell gland persists in late ovoid staged embryos (~150 hpf). **M-P:** By mid organogenesis (~185 hpf), CfSMP12 is expressed diffusely in the head, as well as in the shell gland edge. **Q-T:** In late organogenesis (~196 hpf) veliger embryos, CfSMP12 is detected in the velar lobes, as well as in posterior tissue inside the presumptive shell.

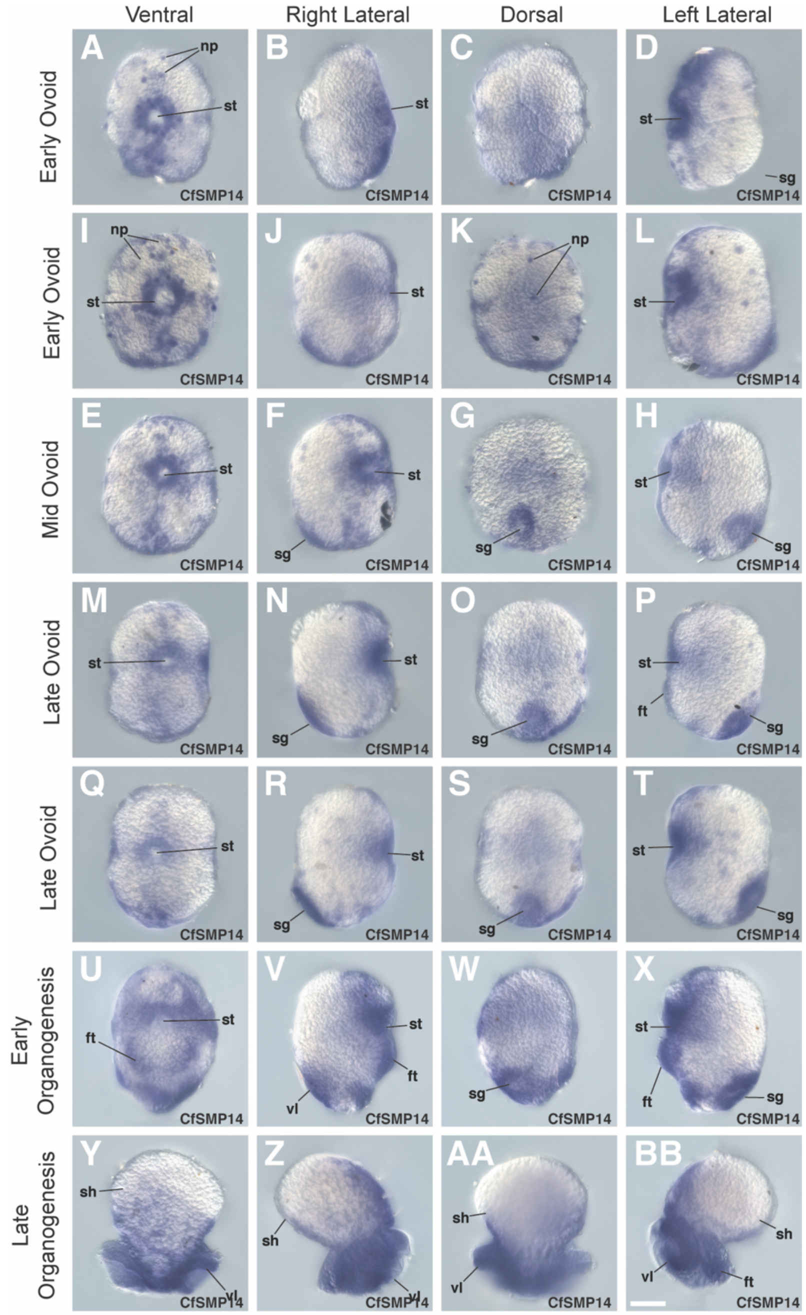

Figure S14: Expression of CfSMP14 during embryogenesis in *C. fornicata.* A-D: CfSMP14 is expressed in the stomodeum and neural precursor cells on the ventral surface during early ovoid stages (~130 hpf). **E-H:** Around 5 hours later (~135 hpf), expression of CfSMP14 persists in cells lining the stomodeum, as well as in ventral neural precursor cells. Expression in dorsal neural precursor cells is also apparent. **I-L:** By mid ovoid stage (~137 hpf), CfSMP14 was detected in the invaginated shell gland, as well as persistent expression in the stomodeum. **M-P:** Shell gland and stomodeum expression continues approximately 8 hours later (~145 hpf) in late ovoid staged embryos. **Q-T:** CfSMP14 continues to be expressed in shell gland and stomodeum during late ovoid stages (~150 hpf). **U-X:** By early organogenesis (~170 hpf), CfSMP14 expression expands from the stomodeum to the foot rudiment, hindgut, and lateral regions of the ventral head. Expression in the shell gland persists. **Y-BB:** CfSMP14 is expressed broadly in the head in veliger larvae (~196 hpf), including the foot, velar lobes, and the anterior regions within the shell.

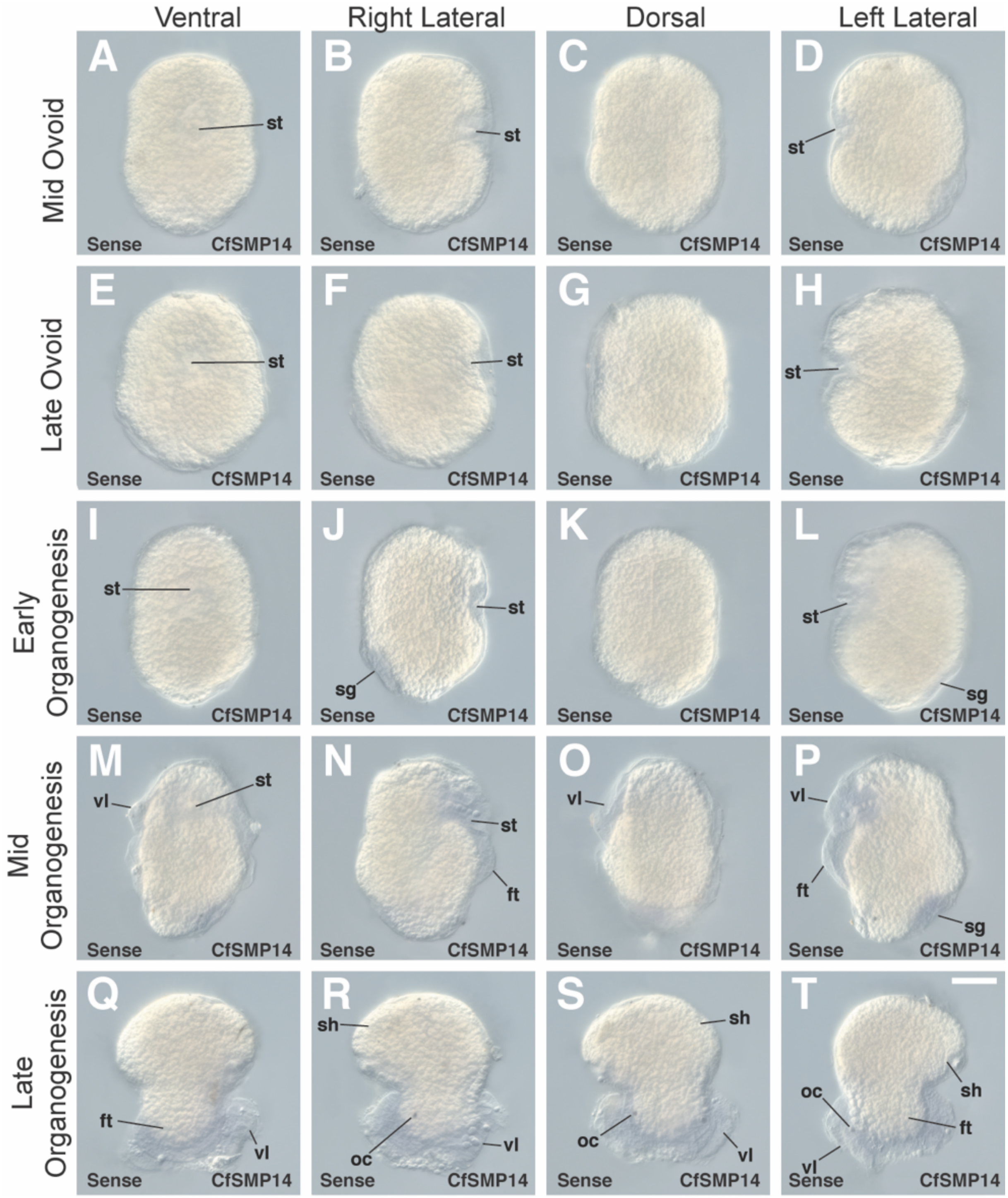

Figure S15: Expression of CfSMP14 Sense during embryogenesis in *C. fornicata.* A-D: Mid ovoid (~140 hpf) staged embryos. **E-H:** Late ovoid staged embryos (~160 hpf). **I-L:** Early organogenesis staged embryos (~170 hpf). **M-P:** Mid organogenesis staged embryos (~180 hpf). **Q-T:** Late organogenesis staged embryos (~196 hpf).

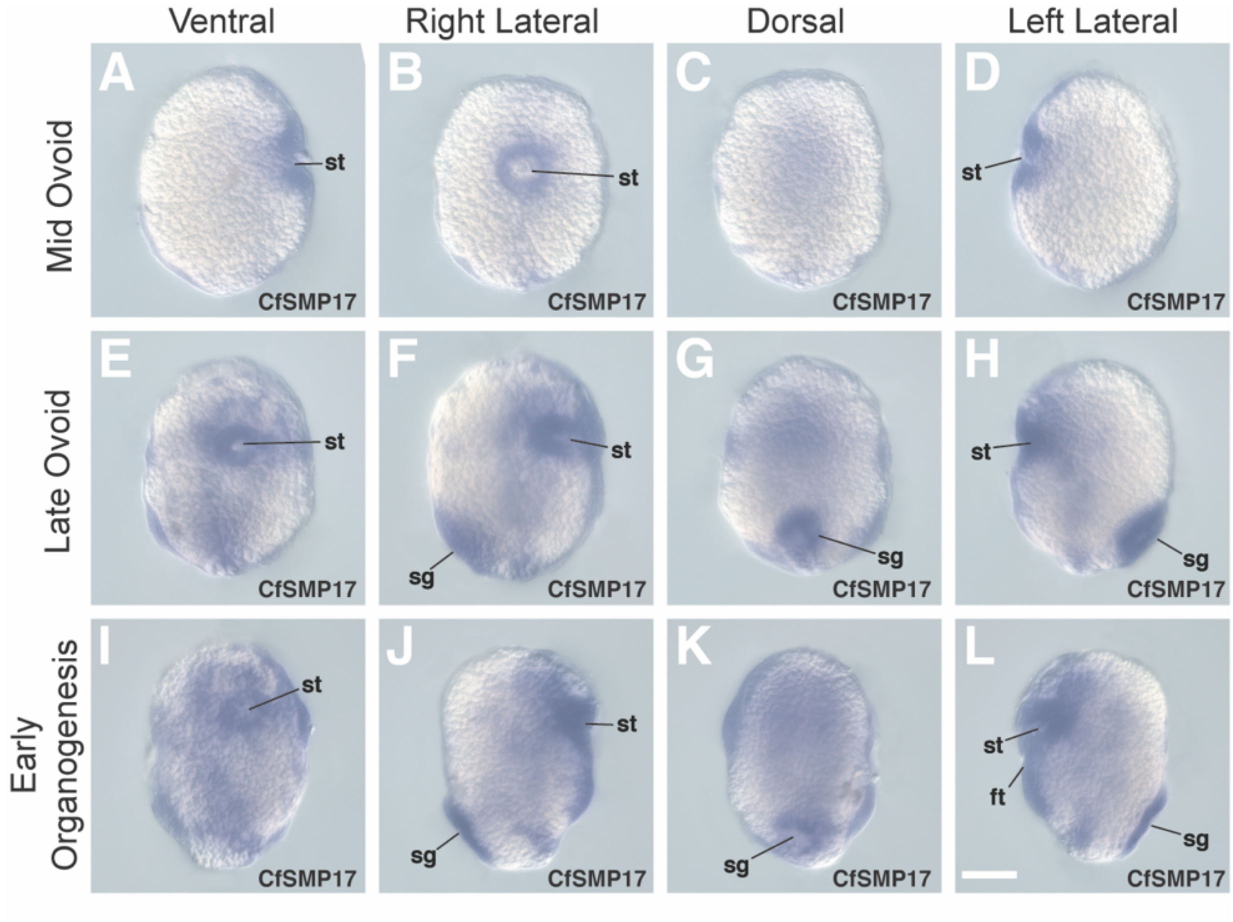

Figure S16: Expression of CfSMP17 during embryogenesis in *C. fornicata.* **A-D:** In mid ovoid (~135 hpf) embryos, CfSMP17 expression is restricted to cells lining the stomodeum. **E-H:** By late ovoid stage (~150 hpf), CfSMP17 is detected in the shell gland, as well as in the stomodeum and ventral surface. **I-L:** During early organogenesis (~170 hpf), CfSMP17 expression persists in the stomodeum and in the shell gland.

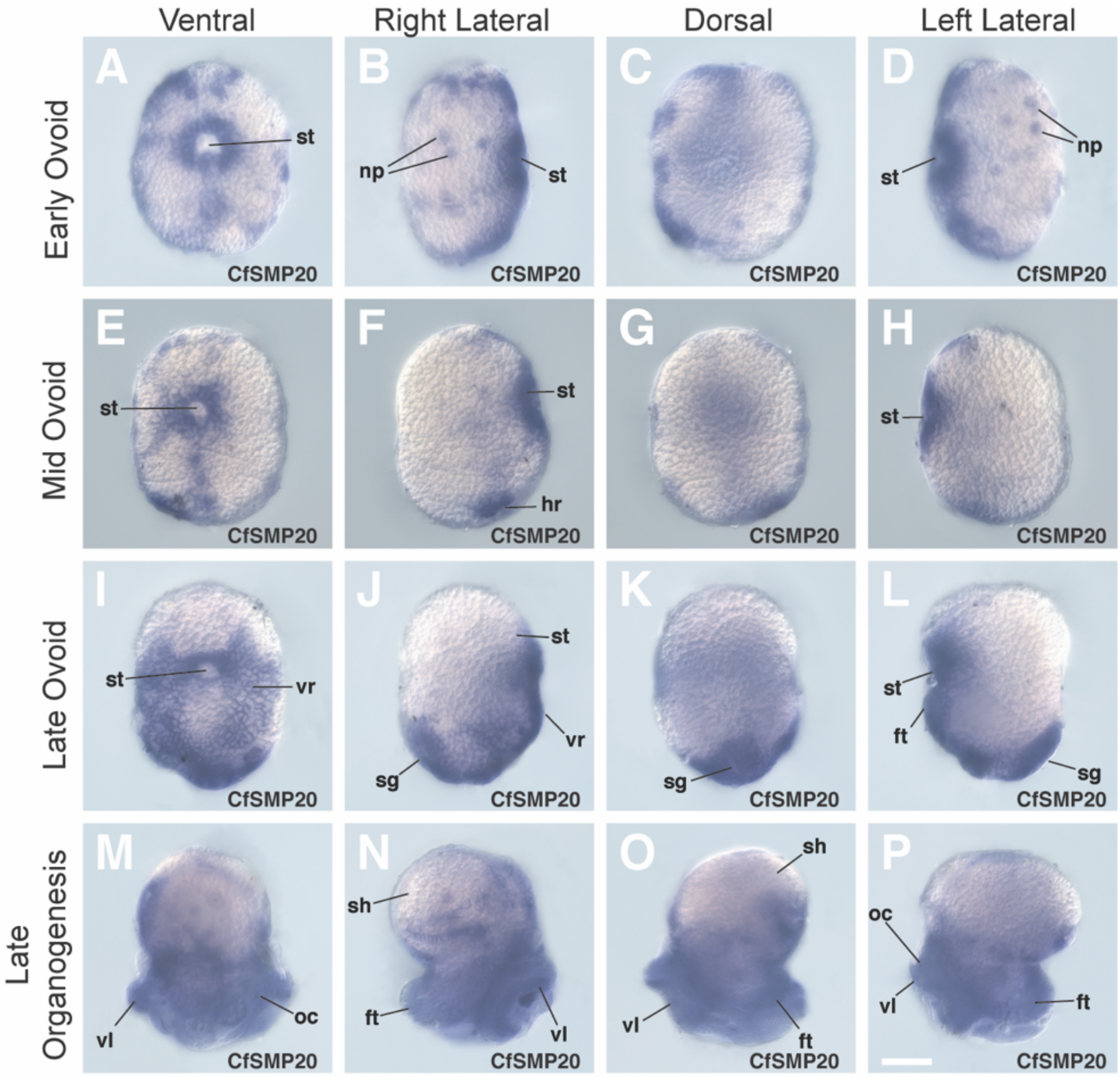

Figure S17: Expression of CfSMP20 during embryogenesis in *C. fornicata.* A-D: Early ovoid (~130 hpf) expression of CfSMP20 is localized to stomodeum and neural precursor cells on the ventral and lateral sides. **E-H:** Mid ovoid expression (~135 hpf) of CfSMP20 is present in the hindgut and stomodeum. **I-J:** In late ovoid (150 hpf) staged embryos, CfSMP20 is expressed in the shell gland, stomodeum and ventral rudiments. **M-N:** By late organogenesis (~196 hpf), CfSMP20 has diffuse expression in the head (including the velar lobes and foot), as well as in epithelial tissues in the shell.

Supplementary Tables

Table S1: Annotation of 97 shell matrix proteins with complete open reading frames in *Crepidula fornicata*

Protein Name	Length (BP)	Annotation	E Value	Accession	Query coverage	Pfam Domains	ID	RLCD	SP	TM	SMP Category
205465	2787	neural-cadherin	0	XP_021379347	73%	Cadherin; TMEM154	Y	-	Y	Y	ECM Binding
211178	2460	EGF, FXa inhibition, SEA, and nido domain-containing protein	0	XP_005107202	99%	EGF; FXa_inhiition; SEA; NIDO	-	Y	-	Y	ECM Binding
260036	1920	sushi, HYR, ephrin receptor-like, and EGF domain-containing protein	0	XP_005107934	84%	HYR; Ephrin_rec_like; Sushi; TSP_1; EGF	Y	-	Y	Y	ECM Binding
138721	1687	klein/chordin-like	1E-09	XP_019620904.1	84%	VWC	-	-	Y	-	ECM Binding
125573	485	Lottia shell protein-17-like	5E-28	B3A0R6	50%	FAM176	-	-	Y	Y	ECM Binding
299504	265	von Willebrand factor C domain-containing protein	2E-52	XP_025092120	74%	VWC	-	-	Y	-	ECM Binding
145408	152	SPARC and WAP domain-containing protein				SPARC_Ca_bdg; WAP	-	-	Y	Y	ECM Binding
14988	364	galaxin-2-like	2E-46	XP_014669156	83%		-	-	Y	-	ECM Binding
34753	364	galaxin-2-like	2E-46	XP_014669156	83%		-	-	Y	-	ECM Binding
145573	344	galaxin-2-like	1E-44	XP_014669156	76%		-	Y	Y	-	ECM Binding
257255	275	reeler domain-containing protein (CfSMP1)	3E-15	RUS86933	95%	Reeler	Y	-	Y	-	ECM Binding
117463	498	Zona pellucida domain-containing protein	7E-30	XP_025081519	68%	Zona_pellucida	Y	-	Y	-	ECM Binding
16558	252	von Willebrand factor C and pacifastin domain-containing protein	8E-08	XP_022082453	47%	VWC; Pacifastin_I	-	-	Y	-	ECM Binding
266962	187	mucin-5AC-like	5E-11	XP_013086696	60%	QVR	-	Y	Y	-	ECM Binding
277483	131	centrosome microtubule binding domain-containing protein				Cep57_MT_bd	-	-	Y	-	ECM Binding
272859	7313	EB module domain-containing protein	0	PVD36172	80%	EB	-	-	Y	Y	ECM Binding
150624	284	EB module domain-containing protein				EB	-	-	Y	-	ECM Binding
250163	286	galactose binding lectin and cartilage oligomeric matrix domain-containing protein	4E-12	PVD33532	44%	Gal_Lectin; COMP	Y	Y	-	-	ECM Binding
163501	1161	chitin binding and immunoglobulin domain containing protein	4E-37	XP_025079776	29%	Ig_3; V-set; I-set; Ig; V-set_CD47; CBM_14	Y	-	-	Y	ECM Binding
300354	681	chitin binding and mucin-17-like domain-containing protein	1E-53	XP_021377427	54%	CBM_14	-	Y	Y	-	ECM Binding
300352	479	chitin binding and mucin-17-like domain-containing protein	1E-53	XP_021377426	77%	CBM_14	-	Y	-	-	ECM Binding
160800	270	lectin L6-like	6E-84	XP_025110889	96%	Hyd_WA	-	-	Y	Y	ECM Binding
267540	243	chitin binding domain-containing protein	6E-63	PVD24918	99%	CBM_14	-	-	Y	-	ECM Binding
126455	215	skin mucus lectin	4E-29	BAE02882	87%	Gal_Lectin	-	-	Y	-	ECM Binding
267406	166	protein obstructor-E-like	4E-55	XP_025092566	99%	CBM_14	-	-	Y	-	ECM Binding
268375	783	polysaccharide deacetylase domain-containing protein	2E-175	XP_021357562	97%	Polysacc_deac_1	Y	Y	Y	-	ECM Binding
284844	425	YajC, Orf78, RCR, EphA2, ECSOR domain-containing protein	7E-15	XP_025100690	76%	YajC; Orf78; RCR; EphA2_TM; ECSCR	Y	-	Y	Y	ECM Binding
80940	377	Actin	0	AJA37852	100%	Actin	-	-	-	-	ECM Binding
322719	150	calmodulin-like	6E-102	CFW94149	99%	EF-hand; SPARC; Caleosin; RNA_pol_Rpb4; TerB; GPHH; Gal_Lectin	-	-	-	-	Ca2+/Signalling
120526	332	calumenin-like	7E-165	XP_025080405	98%	EF-hand; SPARC_Ca_bdg	Y	Y	Y	-	Ca2+/Signalling
80012	317	reticulocalbin-2-like	6E-84	XP_025110417	97%	EF-hand; SPARC_Ca_bdg	Y	-	Y	-	Ca2+/Signalling
94322	205	calmodulin-like	5E-63	XP_025078730	92%	EF-hand; SPARC_Ca_bdg	-	-	Y	Y	Ca2+/Signalling
148591	177	calmodulin-like	2E-13	NP_059118	78%	EF-Hand; SPARC_Ca_bdg	-	-	Y	-	Ca2+/Signalling
109138	150	calmodulin-A-like	1E-29	XP_005092223	99%	EF-hand; SPARC_Ca_bdg	-	-	-	-	Ca2+/Signalling
316786	147	calmodulin-A-like	9E-42	XP_025094746	99%	EF-hand; SPARC_Ca_bdg; S_100	-	-	-	-	Ca2+/Signalling
314074	116	EF-hand and SPARC domain-containing protein				EF-hand; SPARC_Ca_bdg	-	-	Y	-	Ca2+/Signalling
322047	139	fatty acid-binding protein	2E-63	XP_025113673	99%	Lipocalin_7	-	-	-	-	Ca2+/Signalling
80086	222	FK506-binding protein 2-like	1E-95	XP_025094101	100%	FKBP_C; EF-hand	Y	-	Y	-	Ca2+/Signalling
124885	208	ependymin domain-containing protein	1E-07	XP_025088832	99%	Ependymin	-	-	Y	-	Ca2+/Signalling
304585	198	ependymin domain-containing protein	1E-35	XP_025088931	93%	Ependymin	-	-	Y	-	Ca2+/Signalling
206651	144	calmodulin, striated muscle-like	1E-32	XP_012937682	96%	EF-hand; SPARC_Ca_dg	-	-	-	-	Ca2+/Signalling
208205	731	zinc transporter ZIP10	2E-94	XP_011441488.1	37%	Zip	-	Y	Y	Y	Ca2+/Signalling
208211	725	zinc transporter ZIP10	1E-93	XP_011441488.1	37%	Zip	-	Y	Y	Y	Ca2+/Signalling
211993	257	14-3-3 protein epsilon-like	2E-155	XP_025093079	100%	14-3-3	Y	-	-	-	Ca2+/Signalling
214146	405	calreticulin	0	ALY11013	87%	Calreticulin	Y	Y	Y	-	Ca2+/Signalling
238122	273	neural proliferation differentiation and control 1 domain-containing protein	8E-94	XP_025113182	98%	NPDC1	Y	-	Y	Y	Ca2+/Signalling
252967	161	neudesin-like	2E-65	XP_025103507	95%	Cyt-b5	-	-	Y	Y	Ca2+/Signalling
224475	135	Corazonin domain-containing protein				Corazonin	Y	-	Y	-	Ca2+/Signalling
234593	295	meteorin-like protein	3E-112	XP_025101236	100%		-	-	Y	-	Ca2+/Signalling
239624	1481	CD109 antigen-like	4E-157	XP_022294874	97%	A2M; Thiol-ester_cl; SQHop_cyclase_C	-	-	Y	-	Protease/Inhibitors
71080	562	CD109 antigen-like	2E-87	XP_025086512	71%	A2M; Podoplanin; MGC-24; TMEM154	-	-	Y	Y	Protease/Inhibitors
290967	903	reprolysin domain-containing protein	0	XP_025097717	93%	Reprolysin; Pep_M12_propep; Peptidase_M66	Y	Y	Y	Y	Protease/Inhibitors
1967	234	peptidase inhibitor 16-like	2E-57	XP_025110643	86%	CAP	-	-	Y	Y	Protease/Inhibitors
113228	523	Spc7, IncA, Golgin A5 domain-containing protein	2E-25	XP_021345908	54%	Spc7; IncA; Golgin_A5	Y	Y	-	Y	Enzymatic
294303	457	elongation factor 1-alpha-like	0	XP_025090809	100%	GTP_EFTU; GTP_EFTU_D2,3,4; MMR_HSR1; Gtr1_RagA	-	-	-	-	Enzymatic
169471	601	chitinase-like lectin	0	AJA37831	98%	Glyco_hydro_18; CBM_14	-	-	Y	Y	Enzymatic
189669	1020	serine/threonine-protein kinase N2-like	0	XP_025111624	100%	Pkinase; HR1; Pkinase_Tyr; Kinase-like; Uds1	Y	Y	-	-	Enzymatic
138667	990	hephaestin-like protein	0	XP_025095314	97%	Cu-oxidase	-	-	-	-	Enzymatic
202986	508	cysteine protease ATG4D-like protein	2E-05	XP_027593590	19%		-	Y	Y	Y	Enzymatic
196962	499	protein disulfide isomerase	0	AXL95400	95%	Thioredoxin; OST3_OST6; AhpC-TSA; Calsequestrin; Redoxin; ERp29_N; TraF; HyaE	Y	Y	Y	-	Enzymatic
68799	496	adipocyte plasma membrane-associated protein-like	9E-83	XP_025105912		Str_synth; SGL; Arylesterase	-	-	-	-	Enzymatic
294036	357	glutaminyl-peptide cyclotransferase-like	3E-149	XP_025104480	94%	Peptidase_M28	-	-	-	Y	Enzymatic
296945	283	ribonuclease Oy-like	5E-65	XP_025105480	84%	Ribonuclease_T2	-	-	Y	-	Enzymatic
279767	190	2-iminobutanoate/2-iminopropanoate deaminase-like	7E-65	XP_025109837	69%	Ribonuc_L-PSP	-	-	-	-	Enzymatic
223891	157	Cu-Zn superoxide dismutase	7E-65	ABP65325	92%	Sod_Cu	-	-	-	-	Enzymatic
242120	712	adipocyte plasma membrane-associated protein-like	2E-100	XP_025105373	63%	Str_synth; SGL	Y	Y	-	-	Enzymatic
126061	315	putative lineage-restricted protein					-	Y	Y	-	Lineage Restricted
270002	257	putative lineage-restricted protein					Y	Y	Y	-	Lineage Restricted
309863	235	putative lineage-restricted protein					-	Y	-	Y	Lineage Restricted
166764	222	putative lineage-restricted protein					Y	-	Y	Y	Lineage Restricted
319230	212	putative lineage-restricted protein					-	-	Y	-	Lineage Restricted
82338	157	putative lineage-restricted protein					-	-	Y	-	Lineage Restricted
151998	154	putative lineage-restricted protein					-	-	Y	Y	Lineage Restricted
282172	152	putative lineage-restricted protein					-	-	Y	Y	Lineage Restricted
135722	119	putative lineage-restricted protein					-	Y	Y	Y	Lineage Restricted
110712	119	putative lineage-restricted protein					-	-	Y	-	Lineage Restricted
102712	109	putative lineage-restricted protein					-	-	Y	-	Lineage Restricted
226647	88	putative lineage-restricted protein					Y	Y	Y	Y	Lineage Restricted
54214	59	putative lineage-restricted protein					-	-	Y	Y	Lineage Restricted
236200	583	putative lineage-restricted protein					Y	Y	Y	-	Lineage Restricted
302890	421	uncharacterized protein	2E-05	RUS71974.1	39%		-	Y	Y	Y	Uncharacterized
242982	315	uncharacterized protein	5E-71	XP_005112041	67%		-	-	Y	-	Uncharacterized
297467	303	uncharacterized protein	5E-14	XP_025102326	29%		Y	-	Y	-	Uncharacterized
68925	296	uncharacterized protein	2E-44	XP_025080484	90%		-	Y	Y	-	Uncharacterized
321683	290	uncharacterized protein	0.476	XP_005103694	47%		Y	-	Y	-	Uncharacterized
151078	269	uncharacterized protein	3E-09	XP_025102326	74%		-	Y	Y	-	Uncharacterized
151085	269	uncharacterized protein	3E-09	XP_025102326	74%		-	Y	Y	-	Uncharacterized
128713	248	uncharacterized protein	1E-06	XP_001632839	19%		-	Y	-	-	Uncharacterized
71551	226	uncharacterized protein	2E-17	XP_025093758	71%		-	-	Y	-	Uncharacterized
125281	206	uncharacterized protein	3E-06	XP_025095842	36%		Y	Y	Y	-	Uncharacterized
31134	189	uncharacterized protein	2E-33	XP_025089846	92%		-	-	Y	-	Uncharacterized
143973	161	uncharacterized protein	2E-35	XP_025083488	76%		-	-	Y	-	Uncharacterized
95322	147	uncharacterized protein	8E-20	XP_025102187	67%		-	-	Y	Y	Uncharacterized
126276	126	uncharacterized protein	8E-24	XP_025096413	94%		-	-	-	-	Uncharacterized
328141	119	uncharacterized protein	2E-18	XP_002599774	76%		-	-	Y	Y	Uncharacterized
204121	532	uncharacterized protein	0	XP_012938367	94%		-	-	Y	-	Uncharacterized
250988	183	uncharacterized protein	7E-06	XP_013063705	73%		-	Y	Y	Y	Uncharacterized

Table S2: Annotation of 88 shell matrix proteins with partial open reading frames in *Crepidula fornicata*

Protein Name	Length (BP)	ORF	Annotation	E Value	Accession	Query coverage	Pfam Domains	ID	RLCD	SP	TM	SMP Category
154625	405	3 Prime	EGF domain-containing protein	5.16E-57	XP_025081519	82%	EGF	Y	-	-	-	ECM Binding
192307	208	3 Prime	phosphatidylethanolamine-binding protein	1.20E-18	XP_026866971	73%	PBP	Y	-	-	-	ECM Binding
297439	669	5 Prime	fibropellin-1-like	1.96E-81	XP_025107579	59%	DSL; EGF_CA; hEGF; TMEM154; EGF_3; cEGF; EphA2_TM; FXa_inhiition	Y	-	Y	Y	ECM Binding
286175	622	5 Prime	EGF domain-containing protein	1.16E-26	XP_025081519	56%	EGF	-	Y	Y	-	ECM Binding
142228	557	5 Prime	sushi domain-containing protein	1.90E-38	XP_025082219	67%	Sushi	-	Y	-	-	ECM Binding
142223	482	Internal	sushi domain-containing protein	1.45E-38	XP_025082219	78%	Sushi	-	Y	-	-	ECM Binding
100852	96	Internal	Euhadra shell protein-like	1.64E-01	BBD49800	56%		-	-	-	-	ECM Binding
244333	210	5 Prime	WAP domain-containing protein	1.66E-08	PIC30624	86%	WAP	-	-	Y	-	ECM Binding
244335	202	5 Prime	keratin-associated protein 10-7-like	1.81E-01	XP_024662227	98%	WAP	-	-	-	-	ECM Binding
253414	451	Internal	coiled-coil domain-containing protein 1-like	7.90E-28	XP_013066398	75%	COMP	Y	Y	Y	-	ECM Binding
149847	501	3 Prime	extensin-like	8.00E-06	XP_025100309.1	95%		-	Y	-	Y	ECM Binding
242649	267	3 Prime	mucin-5AC-like isoform	1.41E-16	XP_025112691	37%		Y	-	-	-	ECM Binding
253413	461	5 Prime	coiled-coil domain-containing protein 1-like	7.50E-28	XP_013066399	62%	COMP	Y	Y	Y	Y	ECM Binding
108333	762	3 Prime	protein bark beetle-like	1.33E-38	XP_025081993	32%	CUB; CUB_2	-	-	-	-	ECM Binding
112600	409	5 Prime	fn3. and interferind domain-containing protein	1.35E-31	XP_025108927	90%	fn3, Interfer-ind	-	-	Y	-	ECM Binding
87787	945	Internal	MAM and LDL-receptor class A domain-containing protein 2-like	6.62E-56	XP_022794736	66%	MAM; CUB; F5_F8_type_C; CUB_2	Y	-	Y	-	ECM Binding
71173	1274	3 Prime	discoidin domain-containing protein	2.23E-48	AJA37868	12%	F5_F8_type_C; HAP1_N	Y	Y	Y	-	ECM Binding
120976	382	3 Prime	chitin binding and amnionless domain-containing protein	3.22E-18	XP_025106506	52%	CBM_14; Amnionless	Y	-	-	Y	ECM Binding
237853	224	3 Prime	chitin binding domain-containing protein	3.03E-84	PVD24728	88%	CBM_14	-	-	-	-	ECM Binding
211122	243	5 Prime	C-type lectin domain-containing protein	1.00E-18	XP_025107565	53%	Lectin_C	-	-	Y	-	ECM Binding
119563	304	3 Prime	lytic polysaccharide mono-oxygenase, cellulose-degrading domain-containing protein	2.68E-103	XP_025083757	77%	LPMO_10	Y	-	-	Y	ECM Binding
90119	164	3 Prime	lytic polysaccharide mono-oxygenase, cellulose-degrading domain-containing protein	6.13E-45	XP_005110374	89%	LPMO_10	-	-	-	-	ECM Binding
79995	223	Internal	lytic polysaccharide mono-oxygenase, cellulose-degrading domain-containing protein	1.59E-68	XP_011424285	94%	LPMO_10	-	-	-	-	ECM Binding
242653	286	Internal	chondroitin proteoglycan 4 domain-containing protein				CPG4	Y	Y	-	-	ECM Binding
244029	287	3 Prime	tropomyosin domain-containing protein	2.47E-60	XP_025108361	75%	Tropomyosin_1	Y	Y	-	-	ECM Binding
211121	1780	3 Prime	Frizzled and GPS domain-containing protein	0	PVD24840	60%	7tm_2; GPS; Frizzled; 7TM_GPCR_Sra	Y	Y	-	Y	Ca2+/Signalling
150648	483	3 Prime	neurogenic locus notch homolog protein 1-like	6.98E-45	XP_025098671	47%		Y	Y	-	-	Ca2+/Signalling
281435	202	5 Prime	tumor necrosis factor receptor-like protein	7.12E-08	XP_005141746	32%	TNFR_c6; Ephrin_rec_like	Y	-	Y	-	Ca2+/Signalling
191597	174	5 Prime	ubiquitin domain-containing protein	3.99E-119	CBY16339	100%	Ubiquitin; Rad60-SLD; Telomere_Sde2; Methyltrans_RNA; ACT_5	-	Y	-	-	Ca2+/Signalling
267892	152	5 Prime	sugarporin and ABC transporter domain-containing protein				Sugarporin_N; ABC_tran_CTD; NPV_P10	-	-	-	-	Ca2+/Signalling
305876	315	3 Prime	tissue factor pathway inhibitor-like	1.42E-67	XP_020795903	89%	Kunitz_BPTI	-	Y	-	-	Protease/Inhibitors
217830	68	5 Prime	BPTI/Kunitz domain-containing protein-like	6.31E-13	XP_022312259	84%	Kunitz_BPTI	-	-	-	-	Protease/Inhibitors
260629	180	3 Prime	antistasin domain-containing protein	5.68E-24	RUS78970	73%	Antistasin	-	-	-	-	Protease/Inhibitors
169324	337	Internal	alpha-2-macroglobulin domain-containing protein	2.49E-74	XP_025086734	80%	A2M_N_2	Y	-	-	-	Protease/Inhibitors
260402	888	Internal	MAM and LDL-receptor class A domain-containing protein 2-like	2.65E-47	XP_013393211	65%	MAM; CUB; F5_F8_type_C; CUB_2	-	-	-	-	Protease/Inhibitors
178236	623	5 Prime	heat shock cognate 71 kDa protein-like	0	XP_025083839	100%	HSP70; MreB_Ml; FtsA	-	-	-	-	Enzymatic
158017	631	3 Prime	adipocyte plasma membrane-associated protein-like	1.54E-124	XP_025105373	68%	Str_synth; SGL	Y	-	-	-	Enzymatic
125738	156	3 Prime	thioredoxin II	2.26E-33	XP_962263	71%	Thioredoxin; AhpC-TSA; DIM1	Y	-	-	-	Enzymatic
80073	760	3 Prime	putative lineage-restricted protein					Y	Y	-	-	Lineage Restricted
100853	205	3 Prime	putative lineage-restricted protein					Y	-	-	-	Lineage Restricted
236816	200	3 Prime	putative lineage-restricted protein					Y	-	Y	Y	Lineage Restricted
155681	188	3 Prime	putative lineage-restricted protein					-	-	-	-	Lineage Restricted
300370	166	3 Prime	putative lineage-restricted protein					-	-	Y	-	Lineage Restricted
101327	147	3 Prime	putative lineage-restricted protein					-	Y	-	-	Lineage Restricted
151743	130	3 Prime	putative lineage-restricted protein					-	-	Y	-	Lineage Restricted
94783	128	3 Prime	putative lineage-restricted protein					-	Y	-	Y	Lineage Restricted
72620	127	3 Prime	putative lineage-restricted protein					Y	Y	-	-	Lineage Restricted
248320	88	3 Prime	putative lineage-restricted protein					Y	-	Y	Y	Lineage Restricted
100854	84	3 Prime	putative lineage-restricted protein					Y	Y	-	-	Lineage Restricted
100849	178	5 Prime	putative lineage-restricted protein					-	Y	Y	-	Lineage Restricted
290625	170	5 Prime	putative lineage-restricted protein					-	-	Y	-	Lineage Restricted
212735	122	5 Prime	putative lineage-restricted protein					-	-	-	-	Lineage Restricted
236330	112	5 Prime	putative lineage-restricted protein					-	Y	Y	Y	Lineage Restricted
123468	108	5 Prime	putative lineage-restricted protein					Y	-	Y	Y	Lineage Restricted
123469	108	5 Prime	putative lineage-restricted protein					Y	-	Y	Y	Lineage Restricted
123470	108	5 Prime	putative lineage-restricted protein					Y	-	Y	-	Lineage Restricted
79250	94	5 Prime	putative lineage-restricted protein					-	-	Y	-	Lineage Restricted
101039	70	5 Prime	putative lineage-restricted protein					-	Y	Y	Y	Lineage Restricted
160557	50	5 Prime	putative lineage-restricted protein					-	-	-	-	Lineage Restricted
311987	509	Internal	putative lineage-restricted protein					-	Y	Y	-	Lineage Restricted
325029	348	Internal	putative lineage-restricted protein					Y	Y	-	-	Lineage Restricted
158313	340	Internal	putative lineage-restricted protein					Y	Y	-	-	Lineage Restricted
329120	216	Internal	putative lineage-restricted protein					-	-	-	-	Lineage Restricted
128540	213	Internal	putative lineage-restricted protein					-	-	-	-	Lineage Restricted
292533	193	Internal	putative lineage-restricted protein					Y	-	Y	-	Lineage Restricted
201228	166	Internal	putative lineage-restricted protein					-	Y	-	-	Lineage Restricted
100862	96	Internal	putative lineage-restricted protein					-	-	-	-	Lineage Restricted
225023	84	3 Prime	putative lineage-restricted protein					-	-	-	-	Lineage Restricted
263016	611	5 Prime	putative lineage-restricted protein					-	-	-	-	Lineage Restricted
120438	422	5 Prime	putative lineage-restricted protein					-	-	-	Y	Lineage Restricted
217781	291	5 Prime	putative lineage-restricted protein					-	-	Y	-	Lineage Restricted
87323	1201	3 Prime	uncharacterized protein	0	XP_025103188	72%		Y	Y	-	-	Uncharacterized
224843	575	3 Prime	uncharacterized protein	7.01E-11	XP_025113616	35%		Y	Y	-	-	Uncharacterized
117188	476	3 Prime	uncharacterized protein	1.20E-35	XP_025101053	52%		Y	Y	-	-	Uncharacterized
260403	425	3 Prime	uncharacterized protein	1.10E-23	PVD36138	79%		Y	-	-	-	Uncharacterized
11909	370	3 Prime	uncharacterized protein	4.11E-34	XP_025098665	89%		-	-	-	-	Uncharacterized
116784	245	3 Prime	uncharacterized protein	2.42E-30	XP_025108005	50%		-	-	-	Y	Uncharacterized
273880	123	3 Prime	uncharacterized protein	2.08E-20	XP_025082765	97%		-	-	-	-	Uncharacterized
304896	398	5 Prime	uncharacterized protein	1.00E-44	XP_025105448	78%		-	-	-	-	Uncharacterized
306061	146	5 Prime	uncharacterized protein	7.57E-05	XP_013063538	84%			-	-	Y	Uncharacterized
273866	120	5 Prime	uncharacterized protein	4.50E-11	PVD36893	42%		-	Y	Y	-	Uncharacterized
50629	287	Internal	uncharacterized protein	2.10E-06	XP_025079674	64%		-	Y	Y	-	Uncharacterized
285453	192	Internal	uncharacterized protein	3.51E-54	XP_025091093	100%		-	-	-	-	Uncharacterized
163409	191	Internal	uncharacterized protein	3.52E-44	XP_025076260	75%		Y	-	-	-	Uncharacterized
306057	133	Internal	uncharacterized protein	3.03E-04	XP_013063538	80%		-	-	-	Y	Uncharacterized
273872	71	Internal	uncharacterized protein	3.25E-01	PVD36893	48%		-	-	-	-	Uncharacterized
229019	553	Internal	uncharacterized protein	1.76E-107	XP_025086269	93%		-	-	Y	Y	Uncharacterized
163262	409	3 Prime	uncharacterized protein	2.17E-51	XP_025081519	83%		-	-	-	Y	Uncharacterized

Table S3: Summary annotation statistics for shell matrix proteins in *Crepidula fornicata*

Annotation Feature	Complete ORF	5-Prime Partial ORF	3-Prime Partial ORF	Internal Partial ORF	Total	Percent of Proteome
Number of Proteins	97	29	37	22	185	-
BLAST Matches	78	15	25	13	131	71%
Pfam Domains	64	15	15	8	102	55%
Signal Peptide Domains	74	17	5	6	102	55%
Transmembrane Domains	32	8	9	2	51	28%
Repetitive Long Coding Domains	30	8	14	8	60	32%
Intrinsic Disorder	27	6	21	8	62	39%

Table S4: Annotation of 39 differentially expressed mantle genes in *Crepidula fornicata*

Protein Name	SMP ID	Annotation	E Value	Accession	Pfam Domains	ID	SP	TM	ISH	LogFC	LogCPM	Pvalue	FDR
257255	CfSMP1	reeler domain-containing protein	9E-15	RUS86933.1	Reeler	N	Y	Y	Y	4.6119	7.336468014	2.12E-34	4.69E-31
114416		putative lineage-restricted protein				N	N	N	N	4.5848	5.874924689	2.31E-11	4.82E-09
101046		putative lineage-restricted protein				N	N	N	N	4.5553	6.65003782	5.00E-17	2.07E-14
126061	CfSMP2	putative-lineage-restricted protein				Y	Y	Y	Y	4.4617	7.058966771	7.55E-13	1.92E-10
115194		putative lineage-restricted protein				N	N	N	N	4.4058	5.711344962	4.52E-14	1.37E-11
873231	CfSMP3	uncharacterized protein	0	XP_025103196.1		Y	N	Y	Y	4.4047	6.046081199	2.84E-11	5.87E-09
253413	CfSMP4	uncharacterized protein	2E-41	XP_025103196.1		Y	Y	Y	N	4.3691	5.728727921	1.09E-10	2.03E-08
873232	CfSMP5	putative lineage-restricted protein				Y	N	Y	Y	4.3362	7.182903232	1.56E-09	2.50E-07
135297		putative lineage-restricted protein				N	N	N	N	4.3318	5.889802203	3.26E-07	3.57E-05
121701		putative lineage-restricted protein				N	N	N	N	4.2957	5.382988173	8.35E-17	3.41E-14
309864	CfSMP6	eggshell domain-containing protein			Eggshell protein signature	N	Y	Y	N	4.256	5.455724609	4.69E-11	9.32E-09
114417		EGF and zona pelucida-containing protein 1	1E-22	B3A0R6.1	EGF-like; ZP domain	Y	Y	Y	N	4.2495	6.212289447	2.66E-11	5.53E-09
125573	CfSMP7	putative lineage-restricted protein				N	N	Y	N	4.2469	6.622145157	8.91E-07	9.03E-05
300355	CfSMP8	Chitin Binding domain-containing protein	3E-46	AKV63183.1	Chitin Binding Peritrophin-A; Chitin Binding	Y	N	Y	N	4.0992	7.26428096	6.09E-08	7.49E-06
300352	CfSMP9	putative lineage-restricted protein				N	Y	Y	Y	4.0704	4.178178562	9.20E-07	9.27E-05
300354	CfSMP10	putative lineage-restricted protein				N	N	Y	Y	4.0498	6.62555118	2.30E-08	3.02E-06
111368		putative lineage-restricted protein				N	N	Y	N	3.984	4.488040162	0.000382057	0.022520824
236200	CfSMP11	putative lineage-restricted protein				Y	Y	Y	N	3.9514	9.475478041	1.60E-05	0.001295968
141370		putative lineage-restricted protein				N	N	N	N	3.9494	8.22135474	9.25E-05	0.006340613
133617		putative lineage-restricted protein				Y	N	Y	N	3.922	8.22135474	9.25E-05	0.006340613
132767		putative lineage-restricted protein				N	N	N	N	3.8797	7.814793218	0.000117667	0.007873981
127235		putative lineage-restricted protein				N	N	N	N	3.7634	7.715817937	0.000254023	0.015644847
253414	CfSMP12	uncharacterized protein	1E-41	XP_025103203.1		Y	N	Y	Y	3.7016	5.930969505	3.22E-06	0.000292676
877778	CfSMP13	MAM/LDL-receptor domain-containing protein	6E-50	XP_013397766.1	CUB; MAM, memphrin A5/mu	N	Y	Y	N	3.6748	5.461065471	2.95E-05	0.002269445
260629	CfSMP14	fibrillin-1-like	0.000001	XP_033109479.1	Antistasin family	N	N	Y	Y	3.5845	8.127905074	5.34E-07	5.62E-05
309863	CfSMP15	putative lineage-restricted protein				N	N	N	N	3.5004	8.127905074	5.34E-07	5.62E-05
149266		low-density lipoprotein receptor-related protein 2-lik	0	XP_034301274.1	LDL Receptor Class A and B; Calcium-binding EGF	Y	N	Y	N	2.4654	8.318956802	0.000710592	0.038792682
202562	CfSMP16	putative lineage-restricted protein				N	Y	Y	N	2.3822	8.318956802	0.000710592	0.038792682
319230	CfSMP17	putative lineage-restricted protein				N	Y	Y	Y	2.3498	6.949906781	0.000166422	0.010737729
272859	CfSMP18	fibrillin-1-like	0	XP_035824528.1	EGF-like; EB Module	N	Y	Y	N	2.3404	6.831686111	0.000505697	0.028782807
211121	CfSMP19	adhesion G-protein coupled receptor G6-like	0	XP_025107288.1	C Type Lectin; 7 transmembrane receptor	Y	N	Y	N	2.2974	9.417873762	0.001114424	0.057614595
142446		uncharacterized protein	4E-97	XP_009062799.1		N	Y	Y	N	2.0405	10.6792101	0.000467244	0.026828996
116524		putative lineage-restricted protein				N	N	Y	N	1.9234	8.27303193	0.00037042	0.021887756
149198		apoolipoprotein B-100-like	2E-118	XP_013087403.1	Vitellogenin; Von Willebrand Factor D	N	Y	Y	N	1.8263	5.3831867	4.47E-10	7.63E-08
148234		atrial natriuretic peptide receptor 1-like	0	XP_025088267.1	Adenylate Guanylate cyclase; Tyrosine serine/threonine kinase	Y	N	Y	N	1.8206	8.72420945	0.000580964	0.032572691
149208		C type-lectin, CUB, and EGF doomain-containing prote	0	PVD37497.1	CLECT; CUB; EGF; Ephrin receptor-like; Hyr; Sushi; Apple; LDL Rec.	N	Y	Y	N	1.808	7.028623358	0.00064682	0.035779985
109138	CfSMP20	calmodulin-A-like	3E-26	XP_005092223.1	Ef-hand	N	N	Y	Y	1.7774	6.884398742	4.93E-09	7.25E-07
145969		mu-class gst glutathione S-transferase	7E-111	AEI27296.1	Glutathione S-transferase	N	N	Y	N	1.3378	7.743231213	0.001838541	0.088924517
149267		low-density lipoprotein receptor-related protein 2-lik	0	XP_034301274.1	LDL Receptor Class A and B; Calcium-binding EGF	Y	N	Y	N	1.2181	7.670171741	0.519533439	1

Table S5: Primers used to generate riboprobes

Protein Name	SMP ID	Forward Primer	Reverse Primer	Probe Length (bp)
257255	CfSMP1	CATCGCCGACTCCCCACAAACT	GCCCGCACCAAAGTTCTGCATC	634
126061	CfSMP2	GGCAGCTGTGATGGGCCTGA	TGTGGTGGTCCGTGGCTGTG	701
873231	CfSMP3	GGCTGTGGAGGCACTGAAGGTT	AACTGGAAGGGCGAAGACTGGC	719
873232	CfSMP5	GGGAGATCAGGACCGCAGCAAA	AACTACCCCTGTGCCAACGACG	647
300352	CfSMP9	CCACGAGGCCTGCTCTCCAG	CCCCCACCCTGGCTTTCACC	538
300354	CfSMP10	GTGTTGAGGGTAGGGTTGTAGG	ACGAAACTATCTCCACAACCCC	634
253414	CfSMP12	CCACCGCCTGAGAAGACAAGCA	TCGGCCCATCATGGTGATTGCA	1060
260629	CfSMP14	GTAGTGACCCCAAAGCCTCC	GGTGCGATGACTCCGTTGTA	425
319230	CfSMP17	GTTAGCGTGGGGGATGAAGGCA	TGTTGCTCGTGATGGCCATCGT	722
109138	CfSMP20	ACAAGGACATTACCCAACCAGGCT	CGCCAGAGCTCACTGTGACACA	606

Table S6: Multi-tissue assembly statistics

	Transcripts	Genes
**Contig N10**	2516	2675
**Contig N20**	1509	1582
**Contig N30**	999	1035
**Contig N40**	678	728
**Contig N50**	505	544
**Median contig length**	301	309
**Average contig length**	461.25	478.91
**Total assembled bases**	383400791	167641782
**Total sequences**	831218	350048

Table S7: Mantle assembly statistics

	Transcripts	Genes
**Contig N10**	2617	2851
**Contig N30**	1103	1270
**Contig N50**	573	648
**Contig N70**	342	366
**Contig N90**	233	238
**Average contig length**	485.88223	517.71528
**Total assembled bases**	171858487	170831030
**Total sequences**	353704	329971

Supplementary Notes

Supplementary Note 1: Shell Description and Proteome Annotation

Morphological description of adult shells of *C. fornicata*
Crepidula fornicata (Linnaeus 1758), is a caenogastropod mollusc in the family Calyptraeidae. Members of the genus *Crepidula* have limpet-shaped shells with an expanded foot (**Figure S1**). Their shells have a slight convex curve with a posteriorly located apex, with no overt coiling (**Figure S1A-C**). An internal, flattened septum (shelf) attaches to both sides of the shell separating the foot from the visceral mass (**Figure S1A**). The shelf gives the shell the appearance of a slipper, for which its common name "slipper-snail" is derived. On the ventral side of the animal are exposed organs including the gill, foot, head (**Figure S1D-F**). The anterior mantle is visible when viewed ventrally, but extends along the entire shell dorsum (**Figure S1F**).

Extracellular matrix binding proteins
The largest category of SMPs in the shell proteome was extracellular matrix (ECM) binding proteins, comprising 53 of 185 SMPs (**Table S1-S2**). Shell matrix proteins frequently contain binding domains that assist in protein-protein or protein-polysaccharide cross-linking of the extracellular matrix [1]. We annotated an SMP with SPARC (secreted protein, acidic-, cysteine-rich) and a WAP (whey acidic protein) functional domains (314074; **Table S1**). SPARC and WAP domains are cysteine rich and function in polysaccharide-binding [2]. Aragonite-binding SMPs were also recovered, including three SMPs (14988, 34753, 145573; **Table S1**) with similarity to the aragonite-binding protein Galaxin. Additionally, we found four SMPs (237853, 120976, **Table S2**; 163501, 267540, **Table S1**) with carbohydrate-binding module family 14 (CBM_14) domains. CBM 14 domains, also known as peritrophin-A are only present in chitin-binding proteins: chitin is one of the main polysaccharides in the insoluble extracellular matrices in molluscan shells [1]. Two of these SMPs (300354, 300352; **Table S1**) were annotated as mucin-17, which were found surrounding nacre tablets in the mussel, *Pinna nobilis* [3]. Other ECM binding domains include: three von Willebrand factor type C (138721, 299504, 16558; **Table S1**), three Sushi domains (260036, 142228, 142223; **Table S1-S2**), and three epidermal growth factor (EGF) domains (211178, 154625, 286175; **Table S1-2**).

Calcium-binding and signaling proteins
Annotations related to calcium regulation, transport, and binding comprised 26 of 185 SMPs (**Table S1-S2**). The secondary-messenger calmodulin is involved in the calcium-signal transduction pathway, and has been experimentally shown to nucleate aragonite crystals *in-vitro* [4]. Six putative calmodulin-like proteins (322719, 109138, 316786, 206651, 148591, 94322; **Table S1**) were recovered. Calreticulin (214146; **Table S1**) and calumenin (120526; **Table S1**), are both calcium-binding proteins that function in calcium storage [5] and calcium regulation [6], respectively. Calreticulin (214146) was 84% identical to a calreticulin protein from the abalone *Haliotis discus* (ALY11013), while calumenin (120526) was 97% similar to the freshwater snail, *Pomacea canaliculata* (XP_025080405). We recovered a reticulocalbin (80012; **Table S1**) with 97% similarity to a *P. canaliculata* reticulocalbin (XP_025110417). Reticulocalbin is a calcium-binding protein with EF-hand motifs that is also present in chicken eggshells [7]. Ependymin is a calcium-binding glycoprotein that has been reported in many molluscan mantle transcriptomes and shell proteomes [8], including two ependymin-like SMPs in *C. fornicata*'s shell proteome (124885, 304585; **Table S1**). In addition to calcium binding proteins, a number of signaling proteins were identified, including meteorin-like (234593; **Table S1**) a secreted protein involved in skeletal muscle regeneration; a corazonin neuropeptide domain-containing protein (224475; **Table S1**); and a neurogenic notch-like protein (150648; **Table S2**).

Proteases and protease inhibitors

Proteases and protease inhibitors are thought to regulate mineral deposition during biomineralization [1]. Nine SMPs with putative annotations as proteases or protease inhibitors were identified (**Table S1-S2**). For example, we recovered a PI16-like SMP (1967; **Table S1**), which shared 86% sequence similarity to a PI16 sequence from *P. canaliculata*. Peptidase 16 inhibitor (PI16) is a member of a cysteine-rich family of peptidases that inhibit matrix metalloproteinase-2 (MMP-2) [9]. An antistasin-like SMP (260629; **Table S2**), which is an inhibitor of trypsin proteases [10], and an Alpha-2-Macroglobulin (a2M) (169324; **Table S2**), a matrix metalloprotease that has been found in biomineral proteomes of echinoderms [11], were found in the shell proteome. The alpha-2-macroglobulin SMP had 79% sequence similarity and spanned nearly 80% of the complete ORF of a protein sequence from *P. canaliculata*. Two additional SMPs with a2M domains, both with CD109-antigen annotations, were found (71080, 239624; **Table S1**). CD109-antigens are GPI-linked glycoproteins commonly found on human platelets and T-cells, and function in cell-mediated immunity [12]. Finally, two partial SMPs (305876, 217830; **Table S2**), both containing the protease inhibitor domain Kunitz_BPTI (bovine pancreatic trypsin inhibitor) were identified, as well as a putative disintegrin SMP containing four zinc-metalloprotease domains (Reprolysin) with a Peptidase_m66 domain (290967; **Table S1**).

Enzymatic domains and proteins

Enzymatic proteins and domains accounted for 16 of 185 SMPs (**Table S1-S2**). A protein disulfide isomerase (PDI), which catalyzes disulfide bond formations, was identified (196962; **Table S1**). Cysteine bonds are important residues in SMPs due to their thiol groups forming covalent disulfide bonds with other cysteine residues [13]. We recovered another disulfide catalyzing protein (125738; **Table S2**) with similarity to Thioredoxin-II, which in its reduced state, catalyzes the reduction of disulfide bonds [14]. We also identified a chitinase, an enzyme that breaks glycosidic bonds of chitin, and is involved in reshaping chitinous extracellular matrices in molluscs [15] (169471; **Table S1**). This particular chitinase was 98% similar to a chitinase-like lectin from the periwinkle, *Littorina littorea* (AJA37831) and contains a glycoside hydrolase 18 (Glyco_hydro_18) domain, which is responsible for the hydrolysis of glycosidic bonds in chitin [16]. Another enzyme present in the proteome was a ribonuclease Oy (296945; **Table S1**), which was first identified in the oyster *Crassostrea gigas* and is part of the T2 family of ribonucleases that have immune-related functions [17]. Additionally, we found a glutaminyl-peptide cyclotransferase (294036; **Table S4**) which shares 94% amino acid similarity to a glutaminyl-peptide cyclotransferase from *P. canaliculata* (XP_025104480). Finally, we recovered a serine/threonine-protein kinase (189669; **Table S4**) with 99.9% similarity to one found in *P. canaliculata* (XP_025111624).

Lineage-restricted and uncharacterized proteins

Lineage-restricted (31 SMPs) and uncharacterized (34 SMPs) proteins were found in the shell proteome (**Table S1-S2**). The term "lineage-restricted" refers to novel SMPs that have no BLAST hit or Pfam domains [18], whereas the term "uncharacterized" refers to SMPs with BLAST hits, but no Pfam domains [19]. We identified 14 lineage-restricted SMPs with complete ORFs (**Table S4**), and 33 lineage-restricted SMPs with partial ORFs (**Table S2**). Seven of 14 lineage-restricted SMPs with complete ORFs contain regions of intrinsic disorder, and 13 of 14 SMPs have signal peptide sequences (**Table S1)**. Uncharacterized SMPs were split evenly between those containing complete (17 SMPs; **Table S1**) and partial (17 SMPs; **Table S2**) ORFs. We found 26 of 34 uncharacterized SMPs had BLAST hits to *P. canaliculata*. Moreover, 9 of 17 uncharacterized SMPs with complete ORFs had regions of intrinsic disorder, and 15 of 17 SMPs contain signal peptide sequences indicating that they are likely secreted proteins. Recognizing that partial ORFs may contain identifiable domains or regions of similarity once their complete coding sequence is

obtained, we labelled partial SMPs within these two categories, “putative” lineage-restricted or uncharacterized SMPs.

Supplementary Note 2: Annotation of Differentially Expressed Genes

Annotation of 39 differentially expressed genes in the mantle
Twenty of the 39 differentially expressed genes were SMPs previously identified in the shell proteome (**Figure 3D**; **Table S4**). They include 10 SMPs with BLAST hits, and 10 SMPs without BLAST hits (**Table S4; Figure 3D**). The SMPs with BLAST hits had biomineralization related functional domains like chitin binding (CfSMP8; **Table S4**), Epidermal Growth Factor (EGF) (CfSMP18; **Table S4**), Antistasin (CfSMP14; **Table S4**), Reeler (CfSMP1; **Table S4**), and C-type Lectin (CfSMP19; **Table S4**). Two differentially expressed SMPs with clear BLAST hits were for the calcium-signaling gene, calmodulin-A-like (CfSMP20; **Table S4**), as well as one for a memphrin a-5 (MAM) tyrosine-phosphatase/low density lipoprotein receptor (CfSMP13; **Table S4**). Three SMPs (CfSMP3, CfSMP4, CfSMP12) had regions of intrinsic disorder, and had high sequence similarity to uncharacterized proteins from *L. gigantea* (XP_009062799) and *P. canaliculata* (XP_025103196.1) (**Table S4**). Of the 10 differentially expressed SMPs without BLAST hits, 9 had no identifiable protein domains, while one contained an Eggshell domain (CfSMP6; **Table S4**). Three SMPs (CfSMP2, CfSMP5, CfSMP11) without BLAST hits also had extensive regions of intrinsic disorder in their protein coding sequence (**Table S4; Figure 3D**). Lastly, three SMPs (CfSMP9, CfSMP16, CfSMP17) without BLAST hits had signal sequences, but no identifiable domains or regions of intrinsic disorder (**Table S4; Figure 3D**).

Nineteen of 39 differentially expressed genes were not detected in the adult shell proteome of *C. fornicata*, and may perform metabolic or physiological functions in mantle tissue cells (**Figure 3D**; **Table S4**). Eight of 19 differentially expressed genes returned BLAST hits (**Figure 3D**; **Table S4**), including three receptors: one encoding an atrial natriuretic peptide receptor (145969; **Table S4**) that binds natriuretic peptides to mediate extracellular fluid volume [20]; and two low-density lipoprotein receptors that bind lipid carrying lipoproteins [21] (149266, 149266.1; **Table S4**). Interestingly, an apolipoprotein, which is a ligand for LDL receptors was differentially expressed (148234; **Table S4**). Glutathione S-transferase (145969; **Table S4**), an enzyme involved in detoxification of electrophiles within the cytosol of the cell was also differentially expressed [22]. Four genes (114416.1, 149198, 142446, 149208; **Table S4**) had signal sequences, two (149198, 149208) of which contain biomineralization functional domains like Zona pellucida, EGF, Sushi, and C-type lectin. Finally, the remaining 11 of 19 differentially expressed genes had no BLAST hits, or identifiable functional domains, with the exception of 133617 which contains a region of intrinsic disorder.

Supplementary Note 3: Expression of SMPs During Development

Description of shell gland development in *C. fornicata*
In *C. fornicata*, descendants of the second quartet micromeres (2a-d) give rise to the larval shell gland [23,24]. During gastrulation, thickening of the dorsal ectoderm precedes invagination of the shell gland [25,26]. The first visible signs of the invaginated shell gland are apparent at 137 hours post fertilization (hpf), followed by flattening of the shell plate that occurs during organogenesis between 170-196 hpf. Marginal cells surrounding the invaginated shell gland form a “rosette-like ring” around the pore of the invaginated shell gland [26]. Between 196-228 hpf, the invaginated shell gland evaginates to form the shell field, which will expand over the visceral mass to become the mantle epithelium [25–29].

Expression of 8 differentially expressed SMPs during larval shell development
In addition to CfSMP1 and CfSMP2, eight differentially expressed SMPs were screened by *in-situ* hybridization (**Figure S7**). Four of these genes (CfSMP3, CfSMP12, CfSMP14, CfSMP20) had BLAST hits, and were expressed in multiple embryonic tissues including the shell gland (**Figure S7A-P; Table S4**). For example, CfSMP3 is an uncharacterized protein that was expressed in stomodeum, neural precursor cells, and shell gland beginning at mid ovoid stage (150 hpf) (**Figure S7A-B**) and persisting through late ovoid staged embryos (**Figure S7C-D**). CfSMP12 is an uncharacterized protein that was detected in the stomodeum, and had faint shell gland expression in mid ovoid embryos (145 hpf) (**Figure S7E-F**), which became more pronounced by late ovoid stages (**Figure S7G-H**). CfSMP14 is a fibrillin-1-like protein expressed in the shell gland in mid ovoid embryos (145 hpf) (**Figure S7I-J**), which persisted in the shell gland and stomodeum in late ovoid embryos (**Figure S7K-L**). CfSMP20 is a calmodulin A-like protein that was detected in stomodeum and hindgut rudiment in mid (**Figure S7M-N**), and late ovoid embryos (150 hpf) (**Figure S7O-P**). Gene expression patterns were also obtained for four differentially expressed SMPs that did not have BLAST hits (CfSMP5, CfSMP9, CfSMP10, and CfSMP17; **Figure S7Q-FF; Table S4**). Faint shell gland expression of CfSMP5 was apparent in mid ovoid embryos (150 hpf) (**Figure S7Q-R**), and persisted in the same tissues in late ovoid staged embryos (**Figure S7S-T**). CfSMP9 was expressed in the shell gland and stomodeum in mid ovoid staged embryos (160 hpf) (**Figure S7U-V**), and later in the shell gland as well as the foot and stomodeum in late ovoid embryos (**Figure S7W-X**). CfSMP10 was expressed in the shell gland in early ovoid embryos, around 135 hpf (**Figure S7Y-Z**) and persisted in the shell gland in late ovoid embryos (**Figure S7AA-BB**). Lastly, CfSMP17 was expressed in the shell gland and stomodeum starting at mid ovoid (145 hpf) and persisting in the same tissues during late ovoid stages (**Figure S7CC-FF**).

Supplementary Note 4: Methods

Sample preparation for electron and light microscopy
Fracture surfaces were prepared by breaking the shell approximately normal to the anterior-posterior axis. For ground and polished sections, adult shells of *C. fornicata* were embedded in EpoFix resin (Electron Microscopy Sciences) and allowed to cure at RT for 24 hours. Shells were sectioned in a plane normal to the posterior-anterior axis, using a low-speed Isomet™ rotary saw equipped with a diamond blade (Buehler). The resulting sections were ground, in series, with 600, 800, and 1200 grit silicon carbide paper (Buehler). Sections were then polished with 3-μm and 1-μm aqueous diamond suspensions (Buehler), rinsed with DI water, and imaged, or etched for 20 s at RT in 4.3% (w/v) EDTA disodium dihydrate, rinsed again with DI water, and imaged. Light microscope images were taken on a Nikon Eclipse MA200 using crossed-polarized illumination. For electron microscopy, shell fragments and air-dried sections were mounted on aluminum SEM stubs using double-sided carbon tape and coated with 20 nm of Au/Pd using a Denton Desk IV sputter coater (Denton Vacuum). Samples were imaged using a Hitachi SU8030 SEM equipped with a field emission source. Images were recorded at 2 keV acceleration voltage and a working distance of 8.0 mm, using secondary electron contrast.

Sample preparation for powder X-ray diffraction
Approximately 1 g of the shell of each of three adult *C. fornicata* specimens, and of geological aragonite (Top Minerals, Czech Republic) was powdered using mortar and pestle. Powders were analyzed with a voltage of 40 kV and a tube current of 44 mA with a 5 mm slit on a Rigaku Ultima to obtain powder X-ray diffraction (PXRD) data. Background subtraction was performed in CrystalDiffract v6.8.5.

Data processing
Protein identification was performed using a local copy of Mascot (Matrix Science), against a six-frame translation of the mantle transcriptome. Liquid chromatography (LC)-MS/MS data were searched using fixed modification, carbamidomethyl (C) and variable modifications: oxidation (M), acetyl (N-term), pyro-glu (N-term Q), and deamidation (N/Q). Mass values were monoisotopic; peptide mass tolerance was set to 10 ppm; fragment mass tolerance was 0.02 Da; and max missed cleavages was 2. Mascot DAT files were parsed into Scaffold (Proteome Software) for validation, filtering and to create a non-redundant list per sample. Data were filtered using a 95% minimum protein value and 50% minimum peptide value (Prophet scores) requiring at least two unique peptides per protein.

Proteome annotation
Open reading frames were determined using default settings with TransDecoder, and then manually inspected for the presence of at least two peptides inside complete, or partially complete open reading frames. Secreted proteins were searched using SignalP v5.0 [30] for presence of a signal peptide sequence. Transmembrane domains were assessed using THMM v2.0 [31]. Repetitive low complexity domains (RLCDs) and regions of intrinsic disorder were searched using XSTREAM [32] and IUPred [33]. Homology searches were performed using BLASTP against the NCBI-Genbank nr/nt database. Protein domains were identified using hmmer v3.1 against the Pfam database [34].

Whole mount *in situ* hybridization (WMISH)
Embryos of *C. fornicata* were collected and reared at room temperature, followed by fixation in 3.7% paraformaldehyde in filtered seawater (FSW) for 1 h. After fixation, embryos underwent methanol dehydration and were stored at -20˚C as described previously [24,35]. Digoxegenin-labeled riboprobes were made for each SMP gene fragment using a T7 or SP6 MEGAscript kit (Ambion Inc) with DIG-11-UTP (Roche). Embryos were hybridized for a minimum of 48 h at 62˚C using a probe concentration of 1 ng/µl. Probe visualization was achieved using NBT/BCIP color reaction carried out in the dark at room temperature. After WMISH, specimens were washed three times in PTw (1X PBS, 0.5% Tween 20), and cleared in 80% glycerol in 1X PBS. For each gene, 30 embryos of different developmental stages undergoing larval shell gland development were stained. All embryos screened had the same patterns represented in each figure panel.

Supplementary References:

1. Mann S. 2001 *Biomineralization: Principles and Concepts in Bioinorganic Materials Chemistry*. Oxford University Press.

2. Termine JD, Kleinman HK, Whitson SW, Conn KM, McGarvey ML, Martin GR. 1981 Osteonectin, a bone-specific protein linking mineral to collagen. *Cell* 26, 99–105.

3. Marin F, Corstjens P, de Gaulejac B, de Vrind-De Jong E, Westbroek P. 2000 Mucins and molluscan calcification. Molecular characterization of mucoperlin, a novel mucin-like protein from the nacreous shell layer of the fan mussel *Pinna nobilis* (*Bivalvia, pteriomorphia*). *Journal of Biological Chemistry* 275, 20667–20675.

4. Yan Z, Fang Z, Ma Z, Deng J, Li S, Xie L, Zhang R. 2007 Biomineralization: functions of calmodulin-like protein in the shell formation of pearl oyster. *Biochimica et Biophysica Acta* 1770, 1338–1344.

5. Michalak M, Milner RE, Burns K, Opas M. 1992 Calreticulin. *Biochem J* 285 (Pt 3), 681–692.

6. Yabe D, Taniwaki M, Nakamura T, Kanazawa N, Tashiro K, Honjo T. 1998 Human calumenin gene (CALU): cDNA isolation and chromosomal mapping to 7q32. *Genomics* 49, 331–333.

7. Sun C, Xu G, Yang N. 2013 Differential label-free quantitative proteomic analysis of avian eggshell matrix and uterine fluid proteins associated with eggshell mechanical property. *Proteomics* 13, 3523–3536.

8. McDougall C, Hammond MJ, Dailey SC, Somorjai IML, Cummins SF, Degnan BM. 2018 The evolution of ependymin-related proteins. *BMC Evolutionary Biology* 18, 182.

9. Hazell GGJ, Peachey AMG, Teasdale JE, Sala-Newby GB, Angelini GD, Newby AC, White SJ. 2016 PI16 is a shear stress and inflammation-regulated inhibitor of MMP2. *Scientific Reports* 6, 39553.

10. Mittl PR, Di Marco S, Fendrich G, Pohlig G, Heim J, Sommerhoff C, Fritz H, Priestle JP, Grütter MG. 1997 A new structural class of serine protease inhibitors revealed by the structure of the hirustasin-kallikrein complex. *Structure* 5, 253–264.

11. Flores RL, Livingston BT. 2017 The skeletal proteome of the sea star *Patiria miniata* and evolution of biomineralization in echinoderms. *BMC Evolutionary Biology* 17, 125.

12. Lin M, Sutherland DR, Horsfall W, Totty N, Yeo E, Nayar R, Wu X-F, Schuh AC. 2002 Cell surface antigen CD109 is a novel member of the alpha(2) macroglobulin/C3, C4, C5 family of thioester-containing proteins. *Blood* 99, 1683–1691.

13. Zhang C, Zhang R. 2006 Matrix Proteins in the Outer Shells of Molluscs. *Marine Biotechnology*. 8, 572–586.

14. Tanaka T *et al.* 2002 Thioredoxin-2 (TRX-2) is an essential gene regulating mitochondria-dependent apoptosis. *EMBO J.* 21, 1695–1703.

15. Flach J, Pilet PE, Jollès P. 1992 What's new in chitinase research? *Experientia* 48, 701–716.

16. Arakane Y, Muthukrishnan S. 2010 Insect chitinase and chitinase-like proteins. *Cellular and Molecular Life Sciences.* 67, 201–216.

17. Luhtala N, Parker R. 2010 T2 Family ribonucleases: ancient enzymes with diverse roles. *Trends in Biochemical Sciences.* 35, 253–259.

18. Khalturin K, Hemmrich G, Fraune S, Augustin R, Bosch TCG. 2009 More than just orphans: are taxonomically-restricted genes important in evolution? *Trends in Genetics* 25, 404–413.

19. Lubec G, Afjehi-Sadat L, Yang J-W, John JPP. 2005 Searching for hypothetical proteins: theory and practice based upon original data and literature. *Progress in Neurobiology* 77, 90–127.

20. Chinkers M, Garbers DL, Chang MS, Lowe DG, Chin HM, Goeddel DV, Schulz S. 1989 A membrane form of guanylate cyclase is an atrial natriuretic peptide receptor. *Nature* 338, 78–83.

21. Hoofnagle AN, Heinecke JW. 2009 Lipoproteomics: using mass spectrometry-based proteomics to explore the assembly, structure, and function of lipoproteins. *Journal of Lipid Research* 50, 1967–1975.

22. Strange RC, Spiteri MA, Ramachandran S, Fryer AA. 2001 Glutathione-S-transferase family of enzymes. *Mutation Research/Fundamental and Molecular Mechanisms of Mutagenesis* 482, 21–26.

23. Hejnol A, Martindale MQ, Henry JQ. 2007 High-resolution fate map of the snail *Crepidula fornicata*: the origins of ciliary bands, nervous system, and muscular elements. *Developmental Biology.* 305, 63–76.

24. Lyons DC, Perry KJ, Henry JQ. 2015 Spiralian gastrulation: germ layer formation, morphogenesis, and fate of the blastopore in the slipper snail *Crepidula fornicata*. *Evodevo* 6, 24.

25. Hohagen J, Jackson DJ. 2013 An ancient process in a modern mollusc: early development of the shell in *Lymnaea stagnalis*. *BMC Developmental Biology* 13, 27.

26. Kniprath E. 1981 Ontogeny of the Molluscan Shell Field: a Review. *Zoologica Scripta* 10, 61–79.

27. Timmermans LPM. 1968 Studies On Shell Formation in Molluscs. *Netherlands Journal of Zoology*. 19, 413–523.

28. Johnson AB, Fogel NS, Lambert JD. 2019 Growth and morphogenesis of the gastropod shell. *Proceedings of the National Academy of Sciences* 116, 6878–6883.

29. Lesoway MP, Henry JQ. 2019 Twisted Shells, Spiral Cells, and Asymmetries: Evo-Devo Lessons Learned from Gastropods. *Evolutionary Developmental Biology*. 1–18.

30. Armenteros JJA, Tsirigos KD, Sønderby CK, Petersen TN, Winther O, Brunak S, von Heijne G, Nielsen H. 2019 SignalP 5.0 improves signal peptide predictions using deep neural networks. *Nature Biotechnology.* 37, 420–423.

31. Krogh A, Larsson B, von Heijne G, Sonnhammer EL. 2001 Predicting transmembrane protein topology with a hidden Markov model: application to complete genomes. *Journal of Molecular Biology* 305, 567–580.

32. Newman AM, Cooper JB. 2007 XSTREAM: A practical algorithm for identification and architecture modeling of tandem repeats in protein sequences. *BMC Bioinformatics*. 8.

33. Dosztányi Z, Csizmok V, Tompa P, Simon I. 2005 IUPred: web server for the prediction of intrinsically unstructured regions of proteins based on estimated energy content. *Bioinformatics* 21, 3433–3434.

34. Bateman A *et al.* 2004 The Pfam protein families database. *Nucleic Acids Research.* 32, D138–41.

35. Henry JQ, Perry KJ, Martindale MQ. 2010 β-catenin and early development in the gastropod, *Crepidula fornicata*. *Integrative and Comparative Biology.* 50, 707–719.
